# Sex dependency of subconscious visual perception

**DOI:** 10.1186/s13293-025-00754-z

**Published:** 2025-10-06

**Authors:** Zakia Z. Haque, Daniel J. Fehring, Ranshikha Samandra, Oriana Lamoureux, Alexander J. Pascoe, Farshad A. Mansouri

**Affiliations:** Cognitive Neuroscience Laboratory, Department of Physiology, Monash Biomedicine Discovery Institute, Monash University, Melbourne, VIC 3800 Australia

**Keywords:** Sex dependency of cognitive functions, Sub-liminal perception, Sub-conscious perception cued-target detection task, Multimodal interaction

## Abstract

Males are more susceptible to neurodevelopmental cognitive deficits in their perception of visual information. Subliminally-presented visual stimuli might be subconsciously perceived and consequently influence upcoming decisions, however it is still unclear whether subconscious perception differs between males and females. In this study, young adults performed a two-choice target detection task. In the Baseline condition (trials), participants relied only on their ability to detect the target. In the Cued-conscious condition, a visual cue (information) was presented (250 ms) on the same side of an upcoming target and indicated its location (left/right). In the Subliminal-same condition, a briefly presented (~16 ms) cue correctly indicated the target location, however in the Subliminal-opposite condition the cue was shown on the opposite side of the upcoming target and provided incorrect information. Participants’ performance in the Cued-conscious condition was significantly higher than the Baseline and subliminal conditions. In both females and males, performance in the Subliminal-same and Subliminal-opposite conditions was higher and lower than the Baseline condition respectively; indicating that the subliminal cues (information) affected upcoming decisions. However, the effects were significantly larger in males, suggesting males express heightened sensitivity to subliminal visual information, compared to age- and education-matched females. In both males and females, background acoustic stimuli (Music, White noise or Silence) influenced conscious and subconscious visual information processing. Autonomic nervous system activity, assessed through event-related electrodermal activity, was also differentially modulated by supraliminal and subliminal visual information. Our findings indicate remarkable sex dependency in the effects of subliminal visual information on cognitive functions.

**Supplementary Information:**

The online version contains supplementary material available at 10.1186/s13293-025-00754-z.

## Introduction

Males and females express heightened vulnerability to autism spectrum and major depressive disorders, respectively [1, 2], yet the underlying cognitive and neural mechanisms remain uncharacterized. Deficits in visual perception, social behavior and autonomic nervous regulation are frequently found in neurodevelopmental and neuropsychological disorders [3, 4]. In addition to deficits in conscious perception, impairment in processing subliminal (subconscious) stimuli/cues might further exacerbate patients’ deficits in visual processing and particularly social cognition. Contextual factors such as sex, age and multimodal integration between visual and auditory processing might also affect the susceptibility and severity of such impairment. Therefore, it is critically important to study, at fundamental levels, how these contextual factors affect subconscious perception.

## Subconscious perception in neurodevelopmental disorders

Deficits in perceiving social information and communication are commonly observed in neurodevelopmental disorders, and it has been proposed that impaired processing and integration of supraliminal and subliminal visual cues (information) contribute to such deficits [5–7]. Neuropsychological assessment of patients with autism spectrum disorders indicate that whilst their gaze-triggered attentional orienting in response to supraliminal presentation of eye images remains intact, their response to subliminally presented eye images is impaired [5]. These differences in subconscious perception and response to social cues between typically-developing subjects and patients are also accompanied by differences in the activation profile of brain regions, such as the amygdala [5, 8]. In a two-choice forced detection behavioral test involving rapid presentation of face and house images, when the images were presented for a short period (11.7 ms), patients with autism committed more errors, compared to neurotypically developed controls [9]. However, the performance difference between patients and control participants disappeared at longer presentation times (more than 23.4 ms), suggesting that the perceptual threshold (minimum amount of time required for a behavioral effect) may be increased in autism [9]. In another study [10], children (8 and 13 years old) with autism and age-matched neurotypically developed controls were asked to select which of two neutral faces was more ‘friendly’, when one face was preceded by a subthreshold presentation (33 ms) of an anxious face, and the other a subthreshold neutral face. Controls showed a greater shift away from faces paired with anxious target faces than those of the children with autism, suggesting that subthreshold presentations of anxious faces had less impact on the social choices of children with autism than the controls [10]. Patients with autism show deficits in facial affect recognition, which has been linked to their inadequate visual search strategies [11]. In an emotional face priming paradigm in which the participants’ gaze was controlled to remain on the face, neurotypically-developed controls showed an affective priming effect in both supraliminal and subliminal conditions; however, patients with autism spectrum disorders were impaired in facial affect recognition for both supraliminally- and subliminally-presented faces [12]. These studies suggest that impaired processing of both supraliminal and subliminal visual information might contribute to the behavioral deficits in autism, and that these deficits were not necessarily attributable to differences in gaze patterns, as gaze was controlled in some of these studies.

## Sex dependency of conscious and subconscious perception

Previous studies have confirmed cognitive sex differences in the context of various tasks in which processing of supraliminal (consciously perceived) information is required [13–16]. Whether a sex dependency exists in subconscious perception, modulation of upcoming behavior and emotional state by subliminal stimuli, however, remains largely unknown. Significant sex dependency in the onset and severity of symptoms has also been reported in neurodevelopmental disorders [17]. Males appear to be more prone to neurodevelopmental disorders such as autism [1, 2], however the underlying mechanisms of males’ vulnerability or possible protective mechanisms in females remain unknown. Therefore, it is critically important to examine whether subconscious perception of visual stimuli and its consequence on upcoming decisions and autonomic nervous activity inherently differ between females and males.

## Subconscious perception and modulation of behavior

Various priming and masking tasks have been employed to examine whether and how subliminally-presented visual information can influence cognitive functions and emotional state in humans and monkeys [18–26]. In the majority of these tasks, a briefly presented visual cue (around 16–50 ms) is immediately masked and therefore remains consciously unperceivable. Considering the refresh rate of conventional displays, which are used to present and update the visual items, the minimum achievable presentation period (and immediate masking) with these displays would be approximately 16–17 ms. In various task designs, participants report no conscious perception by such a briefly presented visual stimulus, although their behavior or choice might be affected [18, 20–22]. Therefore, the emergence of subliminal (subconscious) perception with minimally presented visual information can be experimentally verified by assessing the consequence of exposure to briefly presented visual cues on the upcoming decision and emotional/affective state [18, 20, 22, 25, 26]. In this context, a stimulus is considered as subliminal, if it is not consciously perceived, but can still modulate upcoming behavior. Previous studies have shown that allocation of attention and previous knowledge might also affect processing and behavioral modulation by subliminally-presented visual stimuli [21, 27]. Therefore, the participants’ knowledge about the task (presence of the subliminal cue, the period in which it is presented (temporal attention) and its spatial location (spatial attention) might affect the behavioral effects of subliminal cues.

## Interaction of auditory and visual stimuli in conscious and subconscious perception

Accumulated evidence indicates that auditory stimuli can significantly affect the perception of visual stimuli [28–30]. The interaction between auditory and visual information processing may occur at the supraliminal and subliminal perceptual level and influence contingent behavior [24, 31]. Background music, including its harmonic and affective aspects, and even white noise might specifically influence multisensory information integration [31–33], however, it is still unclear whether music can enhance the perception of subliminal visual stimuli and their effects on upcoming decisions, and whether its effects are sex dependent. Addressing this question is critical, as music and white noise have been considered as a treatment or adjunct therapeutic measure for various neuropsychological and neurodevelopmental disorders [34–38].

## Current study

**Fig. 1:**
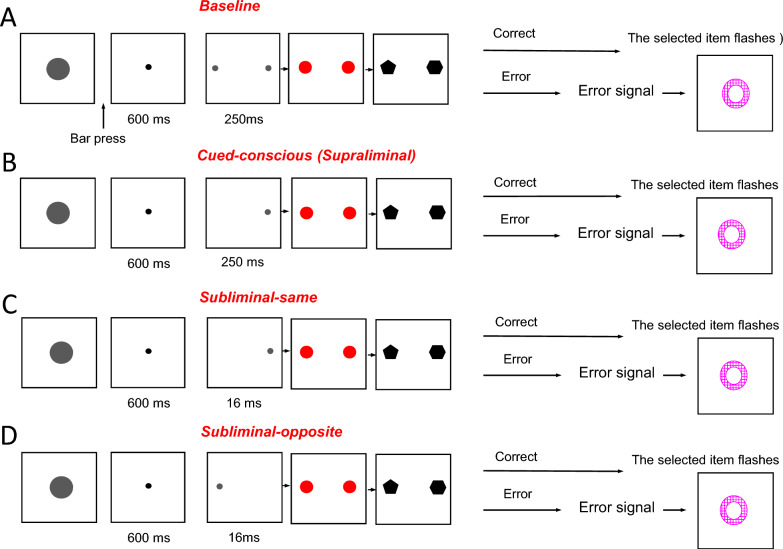
Cued Target Detection Task (cTDT). Four conditions (trials) were included (with the same proportion) in the cTDT. In all conditions, each trial started by presenting a Start-cue (gray filled circle), which instructed participants to press and hold down a switch. After pressing the switch, the Start-cue was replaced by a fixation point at the center of the screen. **A** In the Baseline condition, two small gray circles appeared on the left and right sides of the screen (for 250 ms) and then were replaced by two larger red circles (masks). In all conditions, the masks were presented for 1000, 1300, 1600 or 1900 ms (randomly chosen across trials) and then were replaced by two polygons on the left and right sides of the screen. Participants had to release the switch and touch the polygon with the higher number of sides (target) in the available response window (900 ms). In the Baseline condition, no information regarding the target location was given. **B** In the Cued-conscious condition, the fixation point was replaced by a small gray circle (cue) and remained on the screen for 250 ms. The cue was presented on the left or right side of the screen and indicated the target location. **C** In the Subliminal-same condition, the fixation point was replaced by the same cue, however it was shown only for 16 ms on the left or right side of the screen and indicated the target location. **D** In the Subliminal-opposite condition the cue was shown for 16 ms on the opposite location of the upcoming target. In all conditions, after a correct response, the target flashed two times and then all items disappeared. If participants touched the distractor (the polygon with lower number of sides) or did not respond in the available window, an error signal (a red annulus) was presented for 500 ms. The inter-trial interval was 1400 ms.

In this study, participants performed a cued Target Detection Task (cTDT) (Fig. 1) in which four separate conditions were intermingled. To objectively examine the emergence of subliminal perception, we assessed the modulation of the upcoming decision (participants’ performance) in the Baseline, supraliminal and subliminal conditions. In the cTDT, the Baseline condition indicates the individual ability of each participant in selecting the target in the available response window. We hypothesized that performance in the Cued-conscious condition (where a 250 ms cue was presented on the left or right side of the screen and indicated the target location) will be significantly higher than the Baseline condition. We also hypothesized that performance in the Subliminal-same condition (where a brief (~16 ms) cue was presented on the left or right side of the screen and matched the location of the upcoming target) and in the Subliminal-opposite condition (where a brief (~16 ms) cue was presented on the opposite location of the upcoming target) will be significantly lower than the Cued-conscious condition; because the briefly presented cues would remain below the conscious perception level. Importantly, we hypothesized that performance in the Subliminal-same condition will be higher than the Baseline condition because subliminal information processing might divert attentional resources toward the target location and thus help participants in selecting the target. Conversely, we hypothesized performance in the Subliminal-opposite condition will be lower than the Baseline condition, because subliminal information processing might divert attentional resources away from the target location and thus adversely affect participants’ ability in selecting the target. Therefore, our hypothesis predicted a notable difference in performance between the Subliminal-same and Subliminal-opposite conditions.

We also examined whether background music or white noise can modulate the perception of supraliminal or subliminal visual stimuli (compared to a control, silent condition). Participants were not supposed to pay attention or extract and report any information regarding the background sound, and therefore the background acoustic conditions were irrelevant to the task performance, which required visual information processing in both the supraliminal and subliminal conditions.

Electrodermal activity (EDA) reflects alterations in skin conductance and is mediated through changes in sympathetic aspects of the autonomic nervous system. We measured task-related changes in EDA to gain insight regarding the changes in arousal/emotional state during cognitive task performance and the modulatory effects of supraliminal and subliminal information in various background acoustic conditions. Previous studies [39–41] have shown that, in the context of cognitive tasks, EDA is mainly modulated when the decision outcome becomes evident (around feedback indicating a correct or erroneous decision), and therefore we estimated the maximum task-related change in EDA within a 3-second time interval after a correct or erroneous response [39–41]. Considering the slow development of EDA responses [40, 42, 43], the measurement window for phasic EDA was aligned to the moments after participants received feedback (700 ms post-feedback) about the outcome of their decision in all information conditions. We examined whether the effects of supraliminal and subliminal information on participants’ cognitive functions and autonomic nervous regulation were influenced by background acoustic condition, and whether these were dependent on participants’ sex.

## Methods

### Participants and experimental design

72 University students (40 females) completed a written informed consent form for participating in the study. They were also screened for any past or ongoing neurological or neuropsychological disorders. All participants were third-year university students (either Science, Engineering or Biomedicine) enrolled at the same university. Therefore the participant cohort (males and females) in this study consisted of a homogeneous group of young adults with similar age and education levels. The mean age (± SEM) was 21.0 ± 0.22 years for females and 21.03 ± 0.21 years for males. The age range for both males and females was 19–24 years. All participants, except for two males, were right-handed. All experiments were approved by Monash University Human Research Ethics Committee and all procedures were performed in accordance with the relevant guidelines and regulations. Prior to the day of testing, participants received written instructions regarding the testing and task requirements. Participants were given a verbal briefing outlining the task requirements at the beginning of each testing session. Participants were instructed to perform the cognitive task as accurately and fast as possible, and to select and touch the target (a polygon with the higher number of sides) on a touchscreen in each trial. They were also instructed that there might be a visual cue about the target location in some trials. The presence of subliminal cues was not mentioned in the written or verbal explanations. They then completed practice trials, and data collection commenced once they achieved at least 80% accuracy. Participants performed the cTDT twice in one single session: after performing 200 trials in the pre-rest session, they had between 5-7 minutes rest and then performed the post-rest session (200 trials).

### Cognitive task (Cued Target Detection Task)

The cued Target Detection Task (cTDT) (Fig. 1) included four separate conditions, which were intermingled and randomly presented (with the same proportion). In each trial, participants had to compare the stimuli (left and right polygons) and select the one (target) with the higher number of sides in the available response window (900 ms). The position of the target (left or right) was randomly selected (counterbalanced) across trials. Performance in the Baseline condition (where no information regarding the target location was given) indicated the ability of each participant in selecting the target. In the Cued-conscious condition, a 250 ms cue was presented on the left or right side of the screen and indicated the target location. In the Subliminal-same condition, a brief (~16 ms) cue was presented on the left or right side of the screen and matched the location (left or right) of the upcoming target; however, in the Subliminal-opposite condition a brief (~16 ms) cue was presented on the opposite location of the upcoming target. By inclusion of a Supraliminal-valid condition (Cued-conscious), we encouraged participants to pay attention and detect the imminent but unpredictable cues and use the conveyed information for the upcoming decision. We intentionally did not include Supraliminal-invalid condition (Cued-conscious-opposite) in the task design because after a few experiences with Supraliminal-invalid trials, participants could notice that the presented cue was invalid and then ignore the cues in the rest of the trials. By ignoring the cues, they would not need to be vigilant or pay attention during the trial until the target/distractor appears. This would have led to an unproductive strategy and prevent detecting the effects of supraliminal and also subliminal cues. Participants performed the cTDT in different background acoustic conditions (Silence, White noise, or Music).

#### Hardware and testing setup

We used CORTEX software package (National Institute of Mental Health USA) to control the behavioral task and present the visual stimuli. The computer programs were written/compiled in C language within CORTEX. An input/output board (DAS 16, Keithley) was used to communicate with external systems (response switch and EDA measuring computer at 1 ms temporal resolution). The computer running CORTEX was operated in DOS (Microsoft Disk Operating System) mode, without installing Windows operating system, to avoid any temporal jitter by the resident Windows programs and therefore maximize the temporal resolution. The presentation periods for the supraliminal and subliminal cue, were set at 250 and 10 ms respectively; however, considering the refresh rate of the display (17 inch Microtouch 3M touchscreen) the minimum achievable presentation period for subliminal visual cues was approximately 16 ms. Although a fixation point was presented at the beginning of each trial and participants were instructed to focus on the screen, we did not require head restraint or eye fixation during the task performance. Therefore, the degree of visual angle (DVA) for the images cannot be precisely reported. However, the distance between the participants’ head and the screen was approximately 57 cm. Therefore, for all images, each centimeter on the screen was about 1 DVA. The diameter of the Start-cue (grey circle), Error-signal (purple annulus), Mask (red circle), Cue (small grey circle) and Target/Distractor (white polygons) were 57, 70, 35, 2.5 and 55 millimeters, respectively.

Four researchers (2 males and 2 females) were involved in testing and refining the cognitive task before finalization and use for data collection (which was done in a separate cohort of participants). These 4 researchers individually performed earlier versions of the cognitive task and their subjective reports regarding the perception of the supraliminal and subliminal cues were used to adjust the presentation duration and saliency (size and brightness) of the cues. Furthermore, the response window (900 ms) for selection of the target and delivering the response (releasing the switch and touching the target on the touchscreen) was adjusted according to their performance to maintain correct performance within 60–80% in the Baseline condition.

A response switch was placed in the front-middle of the touchscreen, and participants initiated each trial by pressing and holding down the switch. Participants used their dominant hand’s index finger to press the switch and touch the target on the touchscreen, whilst the surface electrodes for recording EDA were connected to their non-dominant hand, which was supposed to be kept on the desk (without any movement) during task performance. We measured the response time (RT) as the interval between the onset of the polygons (Go-cue) and the switch release at millisecond resolution. Participants used the index finger of their dominant hand to press and release the switch, ensuring a consistent motor response across participants. This design minimized variability due to differences in muscle mass, arm size, or prior motor experience (e.g. musical training). A dedicated response switch, connected directly to a digital input/output board, was used instead of keyboard keys to achieve precise timing resolution. Alternative configurations, such as using left and right hands for separate switches, were avoided to prevent potential confounds from handedness and to reduce motion artifacts in EDA recordings. Similarly, using different fingers of the same hand to operate side-by-side switches could have introduced RT biases due to finger preference or prior experience. Participants performed the cognitive task individually in a dedicated testing room and were monitored through video cameras. Investigators and CORTEX running computers were positioned in a separate control room. The CORTEX computer, running the cognitive tasks, concurrently sent event-related codes (through direct digital input/output connectors) to the EDA measurement system.

### Measuring event-related electrodermal activity (EDA)

An electrodermal recording unit (ML116 GSR Amp- ADInstruments) was used for measuring event-related electrodermal activity (EDA). The EDA amplifier (sampling rate of 75 kHz) was attached to PowerLab (ADI 26T) recording units for the monitoring and storage of data. Skin conductance was continuously recorded by two metal surface electrodes attached to the palmar surface of the index and ring fingers of the non-dominant hand while participants performed the cognitive task. The participants were instructed to keep their non-dominant hand on a pad over the desk and refrain from moving it. Conductivity was measured and reported in Standard International conductance units (microsiemens). Alterations in EDA were monitored in real time and event codes (corresponding to the cognitive task conditions) were automatically sent through the behavioral control software (CORTEX) to allow the assessment of event-related phasic changes in the EDA signal. Amplitude of phasic electrodermal activity was measured as the difference between the maximum and minimum value of the EDA waveform in a 3-second window following each event [40] (Fig. 5C-F). In 13 participants the EDA response could not be reliably recorded in all conditions (due to factors such as motion artefact, cold hands, very low or high levels of sweating), and therefore their data was excluded from the analyses. Before data tabulation and statistical analysis, the raw EDA values were extracted, allowing any issues in the EDA recordings to be identified at this stage (an observer, blind to the analyses and results, extracted and examined the EDA recordings and judged the inclusion or exclusion of data in each participant). Participants had different levels of baseline EDA in the testing session, and therefore to facilitate comparison of the different conditions, EDA values were normalized by dividing the mean EDA in each condition by the mean EDA in all conditions, for each participant.

### Background acoustic conditions

Participants performed the Cued Target Detection Task (cTDT) in various background acoustic conditions (Music, White noise, and Silence). Female and male participants were randomly assigned (counterbalanced) to the three acoustic conditions (between-subject) and performed the cognitive task on a single weekday. Participants wore headphones in all acoustic conditions and the sound level was pre-set for all participants; however, they could also adjust the sound volume according to their preferred level. We have taken this approach to setting sound levels for participants in our previous studies [40, 44]. In the Music condition, classical music without lyrics was randomly selected (with no repetition) and played while participants performed the cognitive task. The White noise condition, generated through Audacity, included all audible frequencies (20 Hz–20 kHz) with the same intensity. In the Silence condition, no sound was played through the headphones. The background sounds were irrelevant to the cognitive task performance and participants could simply ignore them while performing the task.

### Data analyses

We included all collected data points without excluding outliers, because exclusion of any data points requires applying arbitrary criteria. Furthermore, a response window (900 ms) was considered in each trial, and if participants could not complete their response within the response window, the trial was classified as a timeout error. In all trials, the response time (RT) was measured as the time between the onset of the polygons (Go-cue) and the switch release. To ascertain whether there was a sex-dependent difference in subliminal perception in the cTDT performance, participants’ sex was included as a between-subject factor in a repeated-measure ANOVA and applied for each behavioral measure. Each ANOVA contained 4 factors; two within-subject factors: Information (Baseline/Cued-conscious/Subliminal-same/Subliminal-opposite) and Practice (Pre/Post), and two between-subject factors: Sex (Female/Male) and background Acoustic (Silence/Noise/Music). For each within-subject condition, the mean was calculated for each measure in each participant. In this structure, a significant main effect of Information means that participants’ performance was significantly influenced by the type of information given in each trial. The main aim and hypothesis in this study were whether there may be sex-dependent differences in subliminal visual perception, and therefore, in addition to overall analyses including all conditions, we applied an ANOVA only for the Subliminal-same and Subliminal-opposite conditions (in which correct and incorrect information was subliminally conveyed, respectively) in males and females. This comparison could directly examine whether participants’ performance was affected by the fidelity of subliminal visual cues (main effect of Information) and also study the sex dependency of these effects (interaction between Information and Sex factors). When the ANOVA was applied in the two subliminal conditions, a significant two-way interaction between Information and Sex factors would indicate that the subliminal information processing was different between females and males. A significant two-way interaction between Information and Acoustic factors would indicate that the subliminal information processing was affected by the background sound. IBM SPSS statistical package (version 28) was used for statistical analyses. In all analyses, sphericity was confirmed by Mauchly’s test, and if necessary, a Greenhouse-Geisser correction was applied. Partial Eta Squared is reported for all significant effects of each factor (or interactions), and indicates the proportion of the total variance explained by that factor.

## Results

### Subliminally-presented visual information modulated upcoming decisions

#### Percentage of correct responses

The four trial-types (conditions) were intermingled and randomly presented in equal proportion.

**Fig. 2:**
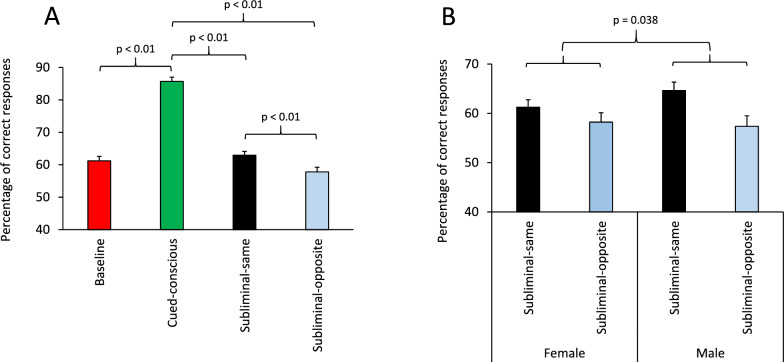
Subliminal (subconscious) perception was sex dependent. **A** The bar-graph shows the percentage of correct responses in different information conditions. In the Baseline condition (red bar), no informative cue was given regarding the target location and therefore performance in the Baseline condition reflects participants’ ability in selecting the target. The highest performance was in the supraliminal (Cued-conscious) condition (green bar). Participants’ performance in the Subliminal-same (black bar) and Subliminal-opposite (blue bar) conditions was higher and lower than the Baseline condition, respectively. Pairwise comparisons (Bonferroni-corrected) indicated a significant differences between the Cued-conscious condition and each of the three other conditions (Baseline, Subliminal-same and Subliminal-opposite). The p value indicates the significance level in each of these pairwise comparisons. Performance of participants in the Subliminal-same condition was significantly higher than the Subliminal-opposite condition. The p value indicates the main effect of Information when an ANOVA directly compared Subliminal-same and Subliminal-opposite conditions. **B** The difference in performance between Subliminal-same and Subliminal-opposite conditions was significantly larger in males compared to the age- and education-matched females. The p value indicates the interaction of the Information and Sex factors in an ANOVA applied in subliminal conditions. In all panels, in all figures, the error bars indicate standard error of the mean (SEM).

We first examined whether the cue (information) provided in each trial affected the participants’ performance. A multi-factor ANOVA [Information (Baseline/Cued-conscious/Subliminal-same/Subliminal-opposite) × Practice (Pre/Post) × Sex (Female/Male) × Acoustic (Silence/Noise/Music)] was applied to the percentage of correct responses. The main effect of Information was highly significant (F(3, 198) = 235.58; p < 0.001) (Partial Eta Squared = 0.78) (Fig. 2A). Pairwise comparisons (Bonferroni-corrected) indicated a significant difference between the Cued-conscious condition and each of the other three conditions (p < 0.01). As hypothesized, performance in the Cued-conscious condition was considerably higher than the other three conditions, indicating that participants significantly benefited from the overt cue in the Cued-conscious condition, but they were not necessarily aware of the presence of subliminal cues. The main effect of Practice or Sex factors was not significant.

To further assess the influence of subliminal information on participants’ performance, we examined whether there was any direct difference between the Subliminal-same and Subliminal-opposite conditions. The trial structure was the same between these two subliminal conditions, with the only difference between them being that cues in the Subliminal-same and Subliminal-opposite conditions conveyed correct and incorrect information about the location of the upcoming target, respectively. We assumed that if subliminal information processing did not occur or could not modulate upcoming decision, there would be no significant difference in performance between the Subliminal-same and Subliminal-opposite conditions; however, if subliminal information processing occurred to the extent that affected participants’ decision making, then there would be a significant difference in performance between these two subliminal conditions. Therefore, we applied a multi-factor ANOVA [Information (Subliminal-same/Subliminal-opposite) × Practice (Pre/Post) × Sex (Female/Male) × Acoustic (Silence/Noise/Music)] only to the two subliminal conditions. The ANOVA showed that the main effect of Information was highly significant (F(1, 66) = 26.23; p < 0.001) (Partial Eta Squared = 0.28): performance in the Subliminal-same condition was significantly higher than the Subliminal-opposite condition, confirming that the correctness of subliminally conveyed information was reflected in participants’ performance (Fig. 2A and supplementary Fig. S1). Importantly, there was a significant interaction between the Information × Sex factors (F(1, 66) = 4.74; p = 0.038) (Partial Eta Squared = 0.063). Figure 2B shows the accuracy of female and male participants in the Subliminal-same and Subliminal-opposite conditions, separately. In both females and males, performance was higher in the Subliminal-same conditions, however the difference between the Subliminal-same and Subliminal-opposite conditions was larger in males (Supplementary Fig. S2 and Table 1). This indicates that subliminal perception occurred in both females and males, however the behavioral effects were more exaggerated in males compared to females. These findings objectively confirm the emergence of subliminal information processing and subsequent modulation of upcoming decisions, and indicate an intriguing sex dependency of subliminal information processing (an increased sensitivity and behavioral modulation by subliminal information in males).

#### Response time

**Fig. 3:**
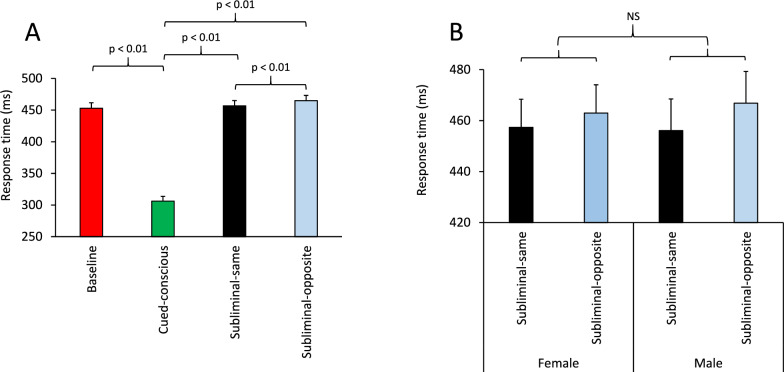
Response time (RT) modulation by supraliminal and subliminal information processing. **A** The bar-graph shows the mean response time in correct trials in various information conditions. The shortest RT was in the Cued-conscious condition, when a supraliminal cue clearly indicated the upcoming target location. Pairwise comparisons (Bonferroni-corrected) indicated significant differences between the Cued-conscious condition and each of the three other conditions (Baseline, Subliminal-same and Subliminal-opposite). The p value indicates the significance level in each of these pairwise comparisons. Participants’ RT in the Subliminal-same condition was significantly shorter than the Subliminal-opposite condition. The p value indicates the main effect of Information when an ANOVA directly compared Subliminal-same and Subliminal-opposite conditions. **B** The difference in RT between Subliminal-same and Subliminal-opposite conditions was comparable (no significant difference - NS) in females and males. The p value indicates the interaction of the Information and Sex factors in an ANOVA applied to RT in subliminal conditions.

To examine whether the information (cue) provided in each trial also influenced the participants’ response time (RT), we applied a multi-factor ANOVA [Information (Baseline/Cued-conscious/Subliminal-same/Subliminal-opposite) × Practice (Pre/Post) × Sex (Female/Male) × Acoustic (Silence/Noise/Music)] to the RT in correct trials. The main effect of Information was highly significant (F(3, 198) = 247.13; p < 0.001) (Partial Eta Squared = 0.79) (Fig. 3A). Pairwise comparisons (Bonferroni-corrected) indicated a significant difference in RT between the Cued-conscious condition and each of the other three conditions (p < 0.01). Response time in the Cued-conscious condition was shorter than the other three conditions, indicating that participants significantly benefited from the overtly conveyed correct information. The main effect of Practice or Sex factors was not significant. To further assess the influence of subliminal information on participants’ RT, we examined whether there was any difference between the Subliminal-same and Subliminal-opposite conditions. We assumed that if subliminal information processing occurred to the extent that affected participants’ decision, then there will be a significant difference in RT between these two subliminal conditions. Therefore, we applied a multi-factor ANOVA [Information (Subliminal-same/Subliminal-opposite) × Practice (Pre/Post) × Sex (Female/ Male) × Acoustic (Silence/Noise/Music)] only to the two subliminal conditions. The ANOVA showed that the main effect of Information was highly significant (F(1, 66) = 14.52; p < 0.001) (Partial Eta Squared = 0.18) (Fig. 3A and supplementary Fig. S3): RT in the Subliminal-same condition was significantly shorter than in the Subliminal-opposite condition. This suggests that the correctness of subliminally conveyed information influenced participants’ RT, and when incorrect information was conveyed in the Subliminal-opposite condition, it adversely affected participants’ RT. There was no significant main effect of the Sex factor (F(1, 66) = 0.006; p = 0.94) (Partial Eta Squared = 0) or its interaction with the Information factor (F(1, 66) = 1.43; p = 0.24) (Partial Eta Squared = 0.021), indicating that the influence of subliminal information on RT was uniformly seen in females and males (Fig. 3B and supplementary Fig. S4).

### Background acoustic stimuli affect conscious and subconscious visual information processing

#### Percentage of correct responses

**Fig. 4:**
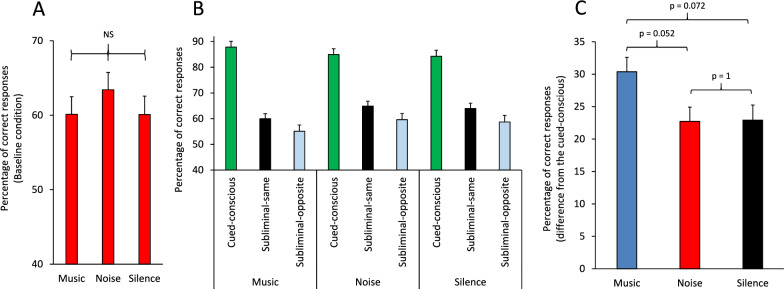
Background acoustic stimuli influenced supraliminal and subliminal perception. **A** The bar-graph shows the percentage of correct responses in the Baseline information condition in the presence of various acoustic stimuli (Silence/Noise/Music). There was no significant difference (NS) in overall performance between these acoustic conditions. **B** The bar-graph shows the percentage of correct responses in supraliminal and subliminal conditions in the presence of various acoustic stimuli. Participants’ performance in supraliminal and subliminal conditions was similar between the Noise and Silence conditions, however they differed in the presence of music. This is better shown in (**C**) where the bar-graph represents the difference in performance between the Cued-conscious (supraliminal) and subliminal conditions for various background acoustic conditions: the difference in performance was larger in the Music condition, compared to the Noise or Silence conditions. Pairwise comparisons (Bonferroni-corrected) indicated a significant difference between Music and Noise conditions (p = 0.052); a near-significant difference between Music and Silence conditions (p = 0.072); but no significant difference between the Noise and Silence conditions (p = 1).

We first examined whether background acoustic conditions (Music, White noise or Silence) had any modulatory effects on participants’ baseline performance by applying a multi-factor ANOVA [Practice (Pre/Post) × Sex (Female/ Male) × Acoustic (Silence/Noise/Music)] to the percentage of correct responses only in the Baseline condition. The ANOVA showed that the main effect of Acoustic condition was not significant (F(2, 66) = 0.66; p = 0.52) (Partial Eta Squared = 0.019), indicating that participants’ performance in the Baseline condition was not different between the three acoustic conditions (Fig. 4A). We then examined how background acoustic conditions influenced supraliminal and subliminal information processing, we applied a multi-factor ANOVA [Information (Cued-conscious/Subliminal-same/Subliminal-opposite) × Practice (Pre/Post) × Sex (Female/ Male) × Acoustic (Silence/Noise/Music)] only to the three cued conditions (the supraliminal and the two subliminal conditions). The main effect of Acoustic condition was not significant, however the interaction between the Information × Acoustic factors was significant (F(2, 66) = 2.59; p = 0.039) (Partial Eta Squared = 0.073). Figure 4B shows that the performance in various information conditions differed between background acoustic conditions. A close examination indicated that the differences in performance between Cued-conscious and subliminal conditions were comparable between the Noise and Silence conditions, however the differences were exaggerated under the Music condition (compared to Noise and Silence conditions). To further assess performance in various acoustic conditions, the performance values in Subliminal-same and Subliminal-opposite conditions were subtracted from the Cued-conscious condition in each Acoustic condition and an ANOVA [Information × Acoustic (Silence/Noise/Music)] was applied to these differences. The main effect of Acoustic factor was significant (F(2, 66) = 3.80; p = 0.027) (Partial Eta Squared = 0.103), indicating that the effects of supraliminal and subliminal cues on participants’ performance were different between the three acoustic conditions (Fig. 4C). Pairwise comparisons (Bonferroni-corrected) indicated a significant difference between Music and Noise conditions (p = 0.052); a near-significant difference between Music and Silence conditions (p = 0.072); but no significant difference between the Noise and Silence conditions (p = 1). These results suggest that, in the Music condition, participants benefited more from the Cued-conscious information, but not as much from the Subliminal-same cue and, in fact, their performance deteriorated more in the Subliminal-opposite condition. When the ANOVA was applied only to the subliminal conditions, the main effect of Acoustic condition or its interaction with the Information factor was not significant. The interaction between Music × Sex factors was not significant in these analyses.

#### Response time

We also applied the multi-factor ANOVA [Practice (Pre/Post) × Sex (Female/Male) × Acoustic (Silence/Noise/Music)] to the RT only in the Baseline condition. The ANOVA showed that the main effect of Acoustic condition was not significant (F(2, 66) = 0.24; p = 0.79) (Partial Eta Squared = 0.007), indicating that participants’ RT in the Baseline condition was not different between the three acoustic conditions. To further examine how background acoustic conditions influenced supraliminal and subliminal information processing, we applied a multi-factor ANOVA [Information (Cued-conscious/Subliminal-same/Subliminal-opposite) × Practice (Pre/Post) × Sex (Female/ Male) × Acoustic (Silence/Noise/Music)] only to RT in the three cued conditions. While the interaction between the Information × Sex × Acoustic factors was significant (F(4, 66) = 4.14; p = 0.003) (Partial Eta Squared = 0.11), the main effect of Acoustic condition was not significant (F(2, 66) = 0.58; p = 0.56) (Partial Eta Squared = 0.17). There was no other significant effect.

### Modulation of autonomic nervous activity

#### Interaction of background acoustic condition and task-relevant information

We measured event-related changes in participants’ EDA as an index of alterations in arousal/emotional state during cognitive task performance and examined the modulatory effects of supraliminal and subliminal information in various background acoustic conditions. Previous studies [15, 39, 45], in the context of executive control tasks, have indicated that the task-related EDA is significantly affected by the outcome of subject’s decision (correct and error) and therefore we aligned the EDA to the feedback (behavioral outcome) given to correct or erroneous decisions.

**Fig. 5:**
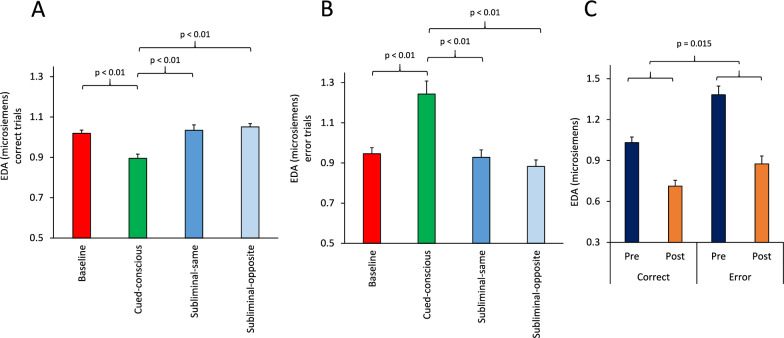
Modulation of electrodermal activity (EDA) by task-related information and context. **A**-**B** The bar-graph shows the mean normalized EDA in correct (**A**) and error (**B**) trials in various information conditions. In correct trials, the smallest EDA was in the Cued-conscious (supraliminal) condition. However, in error trials, the largest EDA was in the Cued-conscious (supraliminal) condition. Please note that in panels **A** and **B**, normalization was done in correct and error trials, separately. The p values indicate the significance of pairwise comparisons (Bonferroni-corrected). **C** The bar-graph shows the mean normalized EDA for correct and error trials in the Pre and Post sessions. The EDA in the Post session was significantly lower than that in the Pre session, indicating a practice-related decline in the EDA. The EDA was significantly larger in error trials. Furthermore, this practice-related decline was larger in error trials compared to the correct trials. The p value indicates the interaction of the Practice and Correctness factors in an ANOVA applied to the mean normalized EDA. Please note that in panel **C**, normalization was done in correct and error trials together, to enable comparing the EDA values between correct and error trials.

We first examined whether EDA was different between various information conditions in correct trials and therefore applied a multi-factor ANOVA [Information (Baseline/Cued-conscious/Subliminal-same/Subliminal-opposite, within-subject) × Practice (Pre/Post, within-subject) × Sex (Female/Male, between-subject) × Acoustic (Silence/Noise/Music, between-subject)] to the normalized EDA values in each condition. There was a significant main effect of Information (F(3,183) = 10.19; p < 0.001) (Partial Eta Squared = 0.14). The lowest EDA was seen in the Cued-conscious condition (Fig. 5A). Pairwise comparisons (Bonferroni-corrected) indicated a significant (p < 0.01) difference between the Cued-conscious condition and each of the other three conditions. These indicated that phasic EDA differed depending on the type of available information for guiding the decision and was lowest when clear information was available for target selection (Fig. 5A). In correct trials, the interaction between Information × Sex factors (F(3,183) = 0.61; p = 0.61) (Partial Eta Squared = 0.01) was not significant. When we applied the ANOVA to normalized EDA in error trials, there was also a significant main effect of Information (F(3,159) = 10.77; p < 0.001) (Partial Eta Squared = 0.17); however, in contrast to EDA modulations in correct trials, the highest EDA was seen in the Cued-conscious condition (Fig. 5B). Pairwise comparisons (Bonferroni-corrected) indicated a significant (p < 0.01) difference between the Cued-conscious condition and each of the other three conditions.

To directly compare the EDA modulations in correct and error trials, we added a factor of ‘Correctness’ to the ANOVA and applied a multi-factor ANOVA [Information (Baseline/Cued-conscious/Subliminal-same/Subliminal-opposite, within-subject) × Practice (Pre/Post, within-subject) × Correctness (Correct/Error, within-subject) × Sex (Female/Male, between-subject) × Acoustic (Silence/Noise/Music, between-subject)] to the normalized EDA values in all correct and error trials. There was a significant main effect of Information (F(3,162) = 3.45; p = 0.018) (Greenhouse-Geiser corrected: F(3,96.9) = 3.45; p = 0.040) (Partial Eta Squared = 0.06), indicating that phasic EDA differed depending on the type of available information for guiding the decision. The main effect of Correctness was also significant (F(1,54) = 56.17; p < 0.001) (Partial Eta Squared = 0.51): the mean EDA was significantly larger in errors, compared to correct trials. The EDA values, for all information conditions, were higher in error trials, suggesting that commission of errors provoked a stronger arousal/emotional response. Additionally, EDA values were different in various information conditions depending on the correctness of the behavior, which was reflected as a significant interaction between Information × Correctness factors (F(3,162) = 20.89; p < 0.001) (Partial Eta Squared = 0.28): in correct trials, the lowest level of EDA was observed in the Cued-conscious condition, whilst in error trials, the largest EDA was in the Cued-conscious condition (Fig. 5A-B). This suggests that in correct trials arousal was at the lowest level when clear (and consciously perceived) information was available for decision making, however committing an error in these trials was unexpected and thus led to the highest arousal level. Supplementary Fig. 5A–D shows representative event-related EDA waveform in one participant: the phasic EDA responses following feedback to correct or error had different magnitudes in various information conditions.

**Fig. 6:**
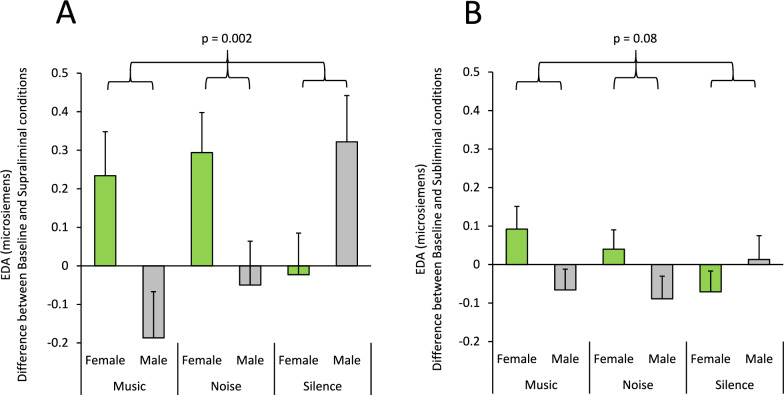
Interactive effects of background acoustic stimuli and participants’ sex in modulating electrodermal activity. **A**–**B** The bar-graph shows the mean normalized EDA in females and males for different background acoustic conditions. **A** The bar-graph shows the difference in EDA between Baseline and supraliminal conditions. In the Silence acoustic condition, the difference in EDA was larger in males, however in the Music and Noise conditions, the differences were much larger in females compared to males. The p value shows the significant interaction between the Acoustic and Sex factors in an ANOVA applied in supraliminal conditions. **B** The bar-graph shows the difference in EDA between Baseline and subliminal conditions. The p value shows that there was no significant interaction between the Acoustic and Sex factors in an ANOVA applied in subliminal conditions.

The main effect of Practice was significant (F(1,54) = 21.65; p = 0.001) (Partial Eta Squared = 0.29): the EDA signal was lower in the post session (Fig. 5C). This suggests that EDA (arousal) response to feedback was significantly decreased after practice. There was also a significant interaction between Correctness × Practice factors (F(1,54) = 6.51; p = 0.014) (Partial Eta Squared = 0.11), suggesting that the rate of practice-related decline in EDA responses was dependent on the correctness of participants’ decision in that trial: the largest practice-related decline was seen in error trials (Fig. 5C). There was also a significant interaction between Information × Acoustic × Sex factors (F(6,162) = 2.89; p = 0.011) (Partial Eta Squared = 0.097). As the interaction of Acoustic and Sex factors may have been related to the type of visual information (supraliminal vs subliminal) in the cued conditions, we further examined whether these interactive effects resulted from alterations in supraliminal and/or subliminal conditions in comparison to the Baseline condition, and therefore we separately applied an ANOVA for supraliminal (Baseline/Cued-conscious) and subliminal (Baseline/Subliminal-same/Subliminal-opposite) conditions. For supraliminal information, the interaction between Information × Acoustic × Sex factors was highly significant (F(2,55) = 6.83; p = 0.002) (Partial Eta Squared = 0.20), however for subliminal information, the interaction was not significant (F(4,122) = 1.85; p = 0.12) (Partial Eta Squared = 0.06). Figure 6 shows the difference in EDA between Baseline and supraliminal conditions (Fig. 6A), and between Baseline and subliminal conditions. An ANOVA applied to these differences indicated a significant interaction between Acoustic and Sex in supraliminal (F(2,55) = 6.82; p = 0.002) (Partial Eta Squared = 0.20), but not in subliminal conditions (F(2,61) = 2.63; p = 0.080) (Partial Eta Squared = 0.08). Suggesting that background acoustic stimuli modulated arousal and emotional state in a sex-dependent manner in the supraliminal condition, but not in the subliminal condition.

## Discussion

In the context of a specifically-designed cognitive task to objectively assess the emergence of subconscious visual perception, we examined the sex dependency of conscious and subconscious visual perception in different background acoustic conditions. Furthermore, we assessed how task-related autonomic regulation was influenced by the interaction between background acoustic stimuli and conscious/subconscious visual information processing. We found that subliminal visual information significantly modulated the upcoming decision: subliminal correct and subliminal incorrect information biased the upcoming decision and led to a higher and lower performance, respectively (Fig. 2A). Importantly, such modulations of the decision process, by subliminally-presented information, was sex-dependent and appeared more pronounced in males, compared to age- and education-matched females (Fig. 2B). Additionally, the effects of supraliminal and subliminal information on the upcoming decision was influenced by the background acoustic condition (Silence/ Noise/ Music): in both sexes, exposure to background music modulated conscious and subconscious visual perception, compared to Silence and Noise conditions (Fig. 4B-C).

We also found concomitant changes in the regulation of the autonomic nervous system, which was reflected as significant alterations in event-related electrodermal activity by various aspects of the cognitive task: (1) EDA was significantly higher after error trials, compared to correct trials (Fig. 5C); (2) there was a practice-related decrease in the EDA in each testing session (Fig. 5C); (3) the EDA was the lowest and highest after consciously perceived visual information in correct and error trials, respectively, when it was compared to Baseline or subliminal conditions (Fig. 5A). These EDA alterations were modulated by the background acoustic stimuli and showed sex dependency. For the supraliminal condition (Fig. 6A), but not for subliminal conditions (Fig. 6B), there was a significant interaction between Acoustic and Sex factors: the difference in EDA modulations between males and females were dependent on the acoustic condition.

### Supraliminal and subliminal information modulated cognitive and autonomic functions

While the cognitive task in this study was developed and refined based on subjective reports and performance of 2 male and 2 female investigators, we objectively assessed the emergence of subliminal perception by examining the behavioral consequence of exposure to correct and incorrect information conveyed by subliminal and supraliminal cues. Critically, participants were not notified about the presence of subliminal cues. The Baseline condition was determined for each participant in which no information to facilitate the upcoming choice was provided. In supraliminal trials (Cued-conscious condition), the visual cue was presented long enough (250 ms) to enable conscious perception, which led to remarkable enhancement of the upcoming decision (highest accuracy and shortest RT) in all participants (Figs. 2A and 3A). Importantly, the subliminal visual cues were presented for the minimum possible duration (for approximately 16 ms and then masked) in Subliminal-same and Subliminal-opposite conditions, in which correct and wrong information were given about the upcoming target location, respectively. We hypothesized that if such subliminal information remains at an unconscious level, performance in these subliminal trials would be significantly lower than the Cued-conscious condition. Critically, we could assess whether the fidelity (correctness) of the subconsciously provided information would translate faithfully to behavioral modulation (i.e., performance in the Subliminal-same condition will be higher than the Subliminal-opposite condition; furthermore, RT in the Subliminal-opposite condition would be longer than the Subliminal-same condition). In fact, participants clearly expressed the predicted behavioral modulations by supraliminal and subliminal visual information (Figs. 2A-B and 3A-B), supporting our hypotheses regarding the emergence of subconscious visual perception in the cTDT.

Previous studies indicate that, in the context of various cognitive tasks with supraliminal stimuli, conflict in information processing adversely affects performance and appears as lower accuracy and longer RT in high-conflict conditions [45–47]. The subliminal cue in the Subliminal-opposite condition conveyed incorrect information regarding the target location, however participants could also consciously compare the two polygons and therefore, a conflict might have arisen between the subliminal and consciously perceived information processing or responses (choices), consequently leading to lower performance and longer RT in the Subliminal-opposite condition (high-conflict), compared to the Subliminal-same (low-conflict) condition. A distributed neural network including the anterior cingulate cortex (ACC), dorsolateral prefrontal cortex (DLPFC) and orbitofrontal cortex (OFC) might be involved in detecting and resolving conflict [45, 47–50]. A previous priming study has reported that conflict between subliminal prime and supraliminal target information affected contingent behavior without concomitant ACC activation, although the ACC was activated when conflict emerged between the supraliminal prime and target [51]. It still remains unclear whether conflict arising from subliminally-conveyed information is processed in the same, or other brain regions to exert its behavioral modulations.

### Sex-dependency of conscious and subconscious perception

*Sex-dependency in cognitive functions*: We found that subconscious modulations of upcoming decisions by subliminal visual cues occurred in both females and males (Fig. 2B), however these modulations appeared more pronounced in males, compared to age- and education-matched females (Fig. 2B). These findings suggest that males are more sensitive to subliminally conveyed information regardless of its correctness and that, compared to females, males demonstrate better performance in the Subliminal-same condition, but performance decreased in the Subliminal-opposite condition. In the context of subliminal priming tasks, where a subliminal affective stimulus was presented before a supraliminal stimulus to examine its influence on affective evaluation of the supraliminal stimulus, a previous study has reported the neural correlate of supraliminal face stimuli (event-related potential N170 in the right hemisphere) was significantly different between males and females [52]. In another study, when fearful faces were subliminally presented, females showed a larger amplitude of the P100 component, compared to men [53]. The P100 component of ERP might reflect low-level visual (including face) information processing and is assumed to be generated in early visual areas. These ERP differences suggest that there might be differences between females and males in initial processing of subliminal visual stimuli [53]. However, in these ERP studies, no difference in behavioral effect of priming was detected, and therefore the significance of these sex-related differences in visual ERP remains to be elucidated.

Previous studies indicate that conscious awareness and attention are closely linked, and allocation of temporal and/or spatial attention might enhance detection and processing of subliminal information and their subsequent effects [21, 54]. In our cTDT, although participants were not aware of the presence of subliminal cues, they could expect cues (on the fixed left or right position; randomly selected across trials) in a known time period within each trial (Fig. 1) and therefore, could allocate their temporal and spatial attention for detecting the cues. In addition, there was a temporal gap between the cue and target onset (Fig. 1), which required remembering the cue location (spatial working memory). Although some past studies suggest that males demonstrate better performance in visuo-spatial attention and spatial working memory tasks, as well as differences in the related brain activation patterns (registered through non-invasive neuroimaging) compared to age-matched females [55–57], others have not found significant differences at the behavioral level [58, 59]. Considering the short interval between the subliminal cues and supraliminal target in our cTDT, the spatial working memory requirement was very limited, and because of a very high performance in Cued-conscious condition, we do not expect that males’ higher sensitivity to subliminal visual stimuli was related to sex differences in spatial working memory. Males’ enhanced sensitivity to visual subliminal information and their behavioral effects in cTDT (Figs. 2–3) might not be necessarily related to their possible advantage in temporal or spatial attention for detecting the cues either, because performance in Baseline and in Cued-conscious conditions were comparable between males and females. Males might have enhanced processing of subliminal information at a more basic, perceptual level, or when it is used for modulating action selection [59, 60]; our results support the former because the overall RT for subliminal and supraliminal stimuli was comparable between females and males. However, males might not have an advantage in using subliminal information for modulating action selection. Such an advantage in perceptual processing of subliminal information might bias allocation of attention and influence choice without enhancing the action selection/execution processes.

*Sex-dependency in autonomic nervous regulation:* We also found that the modulatory effects of supraliminal and subliminal cues on cognitive functions were accompanied by concomitant modulations of autonomic nervous regulation (assessed through measuring event-related electrodermal activity). These modulations were dependent on the correctness of the decision (Fig. 5A-B) and also practice-related learning (Fig. 5C). Importantly, EDA modulations were sex-dependent and influenced by background acoustic condition in supraliminal (Fig. 6A); but, not in subliminal information conditions (Fig. 6B). In the Silence background condition, the difference in EDA between the baseline and supraliminal information condition was higher in males compared to females, however this difference was reversed in Noise and Music background conditions and appeared as higher EDA modulations in females (Fig. 6A). This suggests that the interactive effects of supraliminal and subliminal visual cues and background acoustic stimuli differed between females and males. Previous studies have reported sex differences in autonomic nervous system activity (measuring heart rate and EDA changes) and concomitant changes in activation of brain regions such as the amygdala, insula and ACC (assessed through neuroimaging), in the context of motor and cognitive tasks [61–63]. Our findings are aligned with these past studies and indicate that supraliminally conveyed information might modulate arousal-emotional state and autonomic responses in a sex-dependent manner, which might partly explain the sex-related differences in responses to stressful stimuli and related aberrated autonomic responses in various neuropsychological and neurodevelopmental disorders [64–66].

### Modulation of cognitive and autonomic functions by contextual factors

Our findings suggest that contextual factors such as practice-related learning and background auditory stimuli affect participants’ behavior and their arousal/emotional state (reflected in the EDA). Practice-related behavioral improvement, accompanied by a significant decline in event-related EDA, has also been reported for response inhibition in the context of the Stop-signal task (a test of balance between response execution and action inhibition) [40]. Exposure to background high-tempo music attenuated such practice-related improvements in inhibition ability, but enhanced the EDA decline; however, transcranial direct electrical stimulation (tDCS) over the prefrontal cortex blocked the effects of high-tempo music [40]. Furthermore, it was reported that, while tDCS applied to the prefrontal cortex had no sex-dependent effects on participants’ inhibition ability or response time, the percentage of correct responses was sex-dependently modulated by tDCS [13]. Another previous study has reported significant differences between females and males in benefiting from practice in Stop-signal task (where all visual stimuli, such as Go-cue and Stop-cue, were supraliminally presented): the accuracy in response execution increased, and the ability to inhibit inappropriate actions significantly improved after practice in females, but not in males [15]. Our findings, in the current study, indicate that practice-related changes in arousal-emotional state might affect experience-dependent regulation of autonomic functions in supraliminal and subliminal conditions.

## Limitations

In this study, we used a uniform cohort of males and females in terms of neurotypical development, education (all third year University students) and age range, to enable the examination of the sex dependency of subconscious perception without these potentially confounding factors. Other demographic factors, such as socioeconomic class, and parity, were not registered/controlled. Future studies should examine sex dependency across younger and older age groups, as well as in individuals with neurodevelopmental disorders, and evaluate the influence of other demographic and hormonal factors. Furthermore, our task design only enabled assessing the supraliminal aspects of background acoustic conditions (Silence/ Noise/ Music). Future studies need to examine whether subliminal aspects of these acoustic conditions might also interact with subliminal and supraliminal visual information processing.

## Conclusion

Our findings indicate that subliminal information and contextual factors influence cognitive function and its associated autonomic regulation in a sex-dependent manner. We found that males have a more pronounced response to subliminally-presented cues regardless of the correctness of the conveyed information (i. e. their performance was more affected by both correct and incorrect subliminal information). The implication of males’ pronounced sensitivity to subliminal cues on their susceptibility to neurodevelopmental disorders and associated deficits in social cognition remains unclear, however, our findings suggest that any impairment in subconscious perception might have more severe effects on cognitive abilities in males. The neural substrate and underlying mechanisms of males’ sensitivity to subliminal visual information remains unknown. It is critically important to investigate whether such sex dependency of subconscious perception is linked with differences in incidence, onset time, severity of symptoms and activation of particular brain regions. This might also bring significant insight regarding the susceptibility and underlying neural mechanisms of differences between males and females, who show more susceptibility to autism spectrum and major depressive disorders, respectively.

## Supplementary Information

Additional file 1. Supplementary Figure S1. Subliminal information influenced participants’ accuracy. The bar-graph shows the percentage of correct responses in Subliminal-same and Subliminal-opposite conditions. The p value indicates the main effect of the Information factor in the related ANOVA (when it was applied to the subliminal conditions). The scatterplot (below) displays individual participant accuracy data across both conditions, with the diagonal line indicating equal performance in both. Color-coded mean and standard error are included. The histogram (top right), scaled to the scatterplot, shows the distribution of accuracy differences between conditions, demonstrating a higher accuracy in the Subliminal-same condition.

Additional file 2. Supplementary Figure S2. Subliminal (subconscious) perception was sex dependent. (A) The bar-graph shows the percentage of correct responses in Subliminal-same and Subliminal-opposite conditions, separately for female and male participants. The p value indicates the significance of interaction between Information × Sex factors in the related ANOVA (when it was applied to the subliminal conditions). The scatterplot (bottom) shows individual participant accuracy across both conditions, for both females (blue circles) and males (orange triangles). Accuracy in the Subliminal-same condition was higher, particularly in males.

Additional file 3. Supplementary Figure S3. Subliminal information influenced participants’ response time. The bar-graph shows the response time (RT) in Subliminal-same (Sub-same) and Subliminal-opposite (Sub-opposite) conditions. The scatterplot (below) displays individual participant RT data across both conditions, with the diagonal line indicating equal RT in both. Color-coded mean and standard error are included. The histogram (top right), scaled to the scatterplot, shows the distribution of RT differences between conditions, demonstrating a longer RT in the Subliminal-opposite condition.

Additional file 4. Supplementary Figure S4. No sex dependency was found in the effects of Subliminal information on participants’ response time. (A) The bar-graph shows the Response time (RT) in Subliminal-same and Subliminal-opposite conditions, separately for female and male participants. The p value indicates the significance of interaction between Information × Sex factors in the related ANOVA (when it was applied to the subliminal conditions).The scatterplot (bottom) shows individual participant RT across both conditions, for both females (blue circles) and males (orange triangles).

Additional file 5. Supplementary Figure S5. Representative event-related EDA waveforms in supraliminal and subliminal conditions. (A-D) Representative event-related EDA waveforms in one participant present the phasic EDA responses (microsiemens) following feedback to correct (right panel) or error (left panel) in Baseline (A), Cued-conscious (B), Subliminal-same (C) and Subliminal-opposite (D) conditions. In all panels, the red vertical line indicates the difference between the minimum and maximum points (response amplitude) of the corresponding EDA waveforms.

Additional file 6. Supplementary Table 1. Behavioral and electrodermal activity values in females and males. Table 1 includes the mean ± SE for percentage of correct responses (% correct), Response time (RT) and normalised electrodermal activity for females and males.

## Data Availability

Data is available upon reasonable request to the corresponding author.

## References

[1] Ferri SL, Abel T, Brodkin ES. Sex differences in autism spectrum disorder: a review. Curr Psychiatry Rep. 2018;20(2):9. 10.1007/s11920-018-0874-2. 29504047 10.1007/s11920-018-0874-2 PMC6477922

[2] Walsh MJM, Wallace GL, Gallegos SM, Braden BB. Brain-based sex differences in autism spectrum disorder across the lifespan: a systematic review of structural MRI, fMRI, and DTI findings. NeuroImage: Clin. 2021;31:102719. 10.1016/j.nicl.2021.102719. 34153690 10.1016/j.nicl.2021.102719 PMC8233229

[3] Chung S, Son JW. Visual perception in Autism Spectrum Disorder: A review of neuroimaging studies. J Korean Acad Child Adolesc Psychiatry. 2020;31(3):105–20. 10.5765/jkacap.200018. 10.5765/jkacap.200018 PMC7350544 32665755

[4] Patriquin MA, Hartwig EM, Friedman BH, Porges SW, Scarpa A. Autonomic response in autism spectrum disorder: Relationship to social and cognitive functioning. Biol Psychol. 2019;145:185–97. 10.1016/j.biopsycho.2019.05.004. 31078720 10.1016/j.biopsycho.2019.05.004

[5] Sato W, Kochiyama T, Uono S, Yoshimura S, Toichi M. Neural mechanisms underlying conscious and unconscious gaze-triggered attentional orienting in autism spectrum disorder. Front Hum Neurosci. 2017;11:339. 10.3389/fnhum.2017.00339. 28701942 10.3389/fnhum.2017.00339 PMC5487428

[6] Gaigg SB. The interplay between emotion and cognition in Autism Spectrum Disorder: Implications for developmental theory. Front Integr Neurosci. 2012;6:113. 10.3389/fnint.2012.00113. 23316143 10.3389/fnint.2012.00113 PMC3540960

[7] Hamilton AF. Reflecting on the mirror neuron system in autism: A systematic review of current theories. Dev Cogn Neurosci. 2013;3:91–105. 10.1016/j.dcn.2012.09.008. 23245224 10.1016/j.dcn.2012.09.008 PMC6987721

[8] Zurcher NR, Rogier O, Boshyan J, Hippolyte L, Russo B, Gillberg N, et al. Perception of social cues of danger in autism spectrum disorders. PLoS ONE. 2013;8(12):e81206. 10.1371/journal.pone.0081206. 24324679 10.1371/journal.pone.0081206 PMC3852523

[9] Kleinhans NM, Richards T, Johnson LC, Weaver KE, Greenson J, Dawson G, et al. fMRI evidence of neural abnormalities in the subcortical face processing system in ASD. Neuroimage. 2011;54(1):697–704. 10.1016/j.neuroimage.2010.07.037. 20656041 10.1016/j.neuroimage.2010.07.037 PMC3426450

[10] Hall GB, West CD, Szatmari P. Backward masking: Evidence of reduced subcortical amygdala engagement in autism. Brain Cogn. 2007;65(1):100–6. 10.1016/j.bandc.2007.01.010. 17629385 10.1016/j.bandc.2007.01.010

[11] Berggren S, Engstrom AC, Bolte S. Facial affect recognition in autism, ADHD and typical development. Cogn Neuropsychiatry. 2016;21(3):213–27. 10.1080/13546805.2016.1171205. 27099953 10.1080/13546805.2016.1171205

[12] Kamio Y, Wolf J, Fein D. Automatic processing of emotional faces in high-functioning pervasive developmental disorders: An affective priming study. J Autism Dev Disord. 2006;36(2):155–67. 10.1007/s10803-005-0056-z. 16523242 10.1007/s10803-005-0056-z

[13] Fehring DJ, Samandra R, Haque ZZ, Jaberzadeh S, Rosa M, Mansouri FA. Investigating the sex-dependent effects of prefrontal cortex stimulation on response execution and inhibition. Biol Sex Differ. 2021;12(1):47. 10.1186/s13293-021-00390-3. 34404467 10.1186/s13293-021-00390-3 PMC8369781

[14] Gaillard A, Fehring DJ, Rossell SL. A systematic review and meta-analysis of behavioral sex differences in executive control. Eur J Neurosci. 2021;53(2):519–42. 10.1111/ejn.14946. 32844505 10.1111/ejn.14946

[15] Mansouri FA, Fehring DJ, Gaillard A, Jaberzadeh S, Parkington H. Sex dependency of inhibitory control functions. Biol Sex Differ. 2016;7:11. 10.1186/s13293-016-0065-y. 26862388 10.1186/s13293-016-0065-y PMC4746892

[16] Kheloui S, Jacmin-Park S, Larocque O, Kerr P, Rossi M, Cartier L, et al. Sex/gender differences in cognitive abilities. Neurosci Biobehav Rev. 2023;152:105333. 10.1016/j.neubiorev.2023.105333. 37517542 10.1016/j.neubiorev.2023.105333

[17] Bolte S, Neufeld J, Marschik PB, Williams ZJ, Gallagher L, Lai MC. Sex and gender in neurodevelopmental conditions. Nat Rev Neurol. 2023;19(3):136–59. 10.1038/s41582-023-00774-6. 36747038 10.1038/s41582-023-00774-6 PMC10154737

[18] Elgendi M, Kumar P, Barbic S, Howard N, Abbott D, Cichocki A. Subliminal priming-state of the art and future perspectives. Behav Sci. 2018. 10.3390/bs8060054. 29849006 10.3390/bs8060054 PMC6027235

[19] Meyen S, Zerweck IA, Amado C, von Luxburg U, Franz VH. Advancing research on unconscious priming: When can scientists claim an indirect task advantage? J Exp Psychol Gen. 2022;151(1):65–81. 10.1037/xge0001065. 34264714 10.1037/xge0001065

[20] Dehaene S, Naccache L, Le Clec HG, Koechlin E, Mueller M, Dehaene-Lambertz G, et al. Imaging unconscious semantic priming. Nature. 1998;395(6702):597–600. 10.1038/26967. 9783584 10.1038/26967

[21] Naccache L, Blandin E, Dehaene S. Unconscious masked priming depends on temporal attention. Psychol Sci. 2002;13(5):416–24. 10.1111/1467-9280.00474. 12219807 10.1111/1467-9280.00474

[22] Kouider S, Dehaene S. Levels of processing during non-conscious perception: A critical review of visual masking. Philos Trans R Soc Lond B Biol Sci. 2007;362(1481):857–75. 10.1098/rstb.2007.2093. 17403642 10.1098/rstb.2007.2093 PMC2430002

[23] Sandberg K, Del Pin SH, Overgaard M, Bibby BM. A window of subliminal perception. Behav Brain Res. 2022;426:113842. 10.1016/j.bbr.2022.113842. 35301023 10.1016/j.bbr.2022.113842

[24] Faivre N, Mudrik L, Schwartz N, Koch C. Multisensory integration in complete unawareness: evidence from audiovisual congruency priming. Psychol Sci. 2014;25(11):2006–16. 10.1177/0956797614547916. 25269620 10.1177/0956797614547916

[25] van Vugt B, Dagnino B, Vartak D, Safaai H, Panzeri S, Dehaene S, et al. The threshold for conscious report: signal loss and response bias in visual and frontal cortex. Science. 2018;360(6388):537–42. 10.1126/science.aar7186. 29567809 10.1126/science.aar7186

[26] Ben-Haim MS, Dal Monte O, Fagan NA, Dunham Y, Hassin RR, Chang SWC, et al. Disentangling perceptual awareness from nonconscious processing in rhesus monkeys (Macaca mulatta). Proc Natl Acad Sci U S A. 2021. 10.1073/pnas.2017543118. 33785543 10.1073/pnas.2017543118 PMC8053918

[27] Kiefer M, Brendel D. Attentional modulation of unconscious “automatic” processes: Evidence from event-related potentials in a masked priming paradigm. J Cogn Neurosci. 2006;18(2):184–98. 10.1162/089892906775783688. 16494680 10.1162/089892906775783688

[28] Shams L, Iwaki S, Chawla A, Bhattacharya J. Early modulation of visual cortex by sound: An MEG study. Neurosci Lett. 2005;378(2):76–81. 10.1016/j.neulet.2004.12.035. 15774261 10.1016/j.neulet.2004.12.035

[29] Shams L, Kim R. Crossmodal influences on visual perception. Phys Life Rev. 2010;7(3):269–84. 10.1016/j.plrev.2010.04.006. 20447880 10.1016/j.plrev.2010.04.006

[30] Shams L, Ma WJ, Beierholm U. Sound-induced flash illusion as an optimal percept. NeuroReport. 2005;16(17):1923–7. 10.1097/01.wnr.0000187634.68504.bb. 16272880 10.1097/01.wnr.0000187634.68504.bb

[31] Lin C, Yeh M, Shams L. Subliminal audio-visual temporal congruency in music videos enhances perceptual pleasure. Neurosci Lett. 2022;779:136623. 10.1016/j.neulet.2022.136623. 35398533 10.1016/j.neulet.2022.136623

[32] Bravo F, Glogowski J, Stamatakis EA, Herfert K. Dissonant music engages early visual processing. Proc Natl Acad Sci U S A. 2024;121(30):e2320378121. 10.1073/pnas.2320378121. 39008675 10.1073/pnas.2320378121 PMC11287129

[33] Manjarrez E, Mendez I, Martinez L, Flores A, Mirasso CR. Effects of auditory noise on the psychophysical detection of visual signals: Cross-modal stochastic resonance. Neurosci Lett. 2007;415(3):231–6. 10.1016/j.neulet.2007.01.030. 17276601 10.1016/j.neulet.2007.01.030

[34] Jia R, Liang D, Yu J, Lu G, Wang Z, Wu Z, et al. The effectiveness of adjunct music therapy for patients with schizophrenia: A meta-analysis. Psychiatry Res. 2020;293:113464. 10.1016/j.psychres.2020.113464. 33002835 10.1016/j.psychres.2020.113464

[35] Ramaswamy M, Philip JL, Priya V, Priyadarshini S, Ramasamy M, Jeevitha GC, et al. Therapeutic use of music in neurological disorders: A concise narrative review. Heliyon. 2024;10(16):e35564. 10.1016/j.heliyon.2024.e35564. 39220936 10.1016/j.heliyon.2024.e35564 PMC11365335

[36] Matziorinis AM, Koelsch S. The promise of music therapy for Alzheimer’s disease: A review. Ann N Y Acad Sci. 2022;1516(1):11–7. 10.1111/nyas.14864. 35851957 10.1111/nyas.14864 PMC9796133

[37] Kaneko Y, Butler JP, Saitoh E, Horie T, Fujii M, Sasaki H. Efficacy of white noise therapy for dementia patients with schizophrenia. Geriatr Gerontol Int. 2013;13(3):808–10. 10.1111/ggi.12028. 23819634 10.1111/ggi.12028

[38] Soderlund GB, Sikstrom S, Loftesnes JM, Sonuga-Barke EJ. The effects of background white noise on memory performance in inattentive school children. Behav Brain Funct. 2010;6:55. 10.1186/1744-9081-6-55. 20920224 10.1186/1744-9081-6-55 PMC2955636

[39] Fehring DJ, Samandra R, Rosa MG, Mansouri FA. Negative emotional stimuli enhance conflict resolution without altering arousal. Front Hum Neurosci. 2019;13:282. 10.3389/fnhum.2019.00282. 31456675 10.3389/fnhum.2019.00282 PMC6700260

[40] Mansouri FA, Acevedo N, Illipparampil R, Fehring DJ, Fitzgerald PB, Jaberzadeh S. Interactive effects of music and prefrontal cortex stimulation in modulating response inhibition. Sci Rep. 2017;7(1):18096. 10.1038/s41598-017-18119-x. 29273796 10.1038/s41598-017-18119-x PMC5741740

[41] Mansouri FA, Buckley MJ, Fehring DJ, Tanaka K. The role of primate prefrontal cortex in bias and shift between visual dimensions. Cereb Cortex. 2020;30(1):85–99. 10.1093/cercor/bhz072. 31220222 10.1093/cercor/bhz072 PMC7029686

[42] Bechara A, Damasio H, Damasio AR, Lee GP. Different contributions of the human amygdala and ventromedial prefrontal cortex to decision-making. J Neurosci. 1999;19(13):5473–81. 10.1523/JNEUROSCI.19-13-05473.1999. 10377356 10.1523/JNEUROSCI.19-13-05473.1999 PMC6782338

[43] Boucsein W, Fowles DC, Grimnes S, Ben-Shakhar G,roth WT, Dawson ME, et al. Publication recommendations for electrodermal measurements. Psychophysiology. 2012;49(8):1017–34. 10.1111/j.1469-8986.2012.01384.x. 22680988 10.1111/j.1469-8986.2012.01384.x

[44] Fehring DJ, Illipparampil R, Acevedo N, Jaberzadeh S, Fitzgerald PB, Mansouri FA. Interaction of task-related learning and transcranial direct current stimulation of the prefrontal cortex in modulating executive functions. Neuropsychologia. 2019;131:148–59. 10.1016/j.neuropsychologia.2019.05.011. 31100345 10.1016/j.neuropsychologia.2019.05.011

[45] Mansouri FA, Egner T, Buckley MJ. Monitoring demands for executive control: Shared functions between human and nonhuman primates. Trends Neurosci. 2017;40(1):15–27. 10.1016/j.tins.2016.11.001. 27986294 10.1016/j.tins.2016.11.001

[46] Mansouri FA, Tanaka K, Buckley MJ. Conflict-induced behavioral adjustment: A clue to the executive functions of the prefrontal cortex. Nat Rev Neurosci. 2009;10(2):141–52. 10.1038/nrn2538. 19153577 10.1038/nrn2538

[47] Kerns JG, Cohen JD, MacDonald AW 3rd, Cho RY, Stenger VA, Carter CS. Anterior cingulate conflict monitoring and adjustments in control. Science. 2004;303(5660):1023–6. 10.1126/science.1089910. 14963333 10.1126/science.1089910

[48] Kuwabara M, Mansouri FA, Buckley MJ, Tanaka K. Cognitive control functions of anterior cingulate cortex in macaque monkeys performing a Wisconsin card sorting test analog. J Neurosci. 2014;34(22):7531–47. 10.1523/JNEUROSCI.3405-13.2014. 24872558 10.1523/JNEUROSCI.3405-13.2014 PMC4035517

[49] Mansouri FA, Buckley MJ, Tanaka K. Mnemonic function of the dorsolateral prefrontal cortex in conflict-induced behavioral adjustment. Science. 2007;318(5852):987–90. 10.1126/science.1146384. 17962523 10.1126/science.1146384

[50] Mansouri FA, Buckley MJ, Tanaka K. The essential role of primate orbitofrontal cortex in conflict-induced executive control adjustment. J Neurosci. 2014;34(33):11016–31. 10.1523/JNEUROSCI.1637-14.2014. 25122901 10.1523/JNEUROSCI.1637-14.2014 PMC4131015

[51] Dehaene S, Artiges E, Naccache L, Martelli C, Viard A, Schurhoff F, et al. Conscious and subliminal conflicts in normal subjects and patients with schizophrenia: The role of the anterior cingulate. Proc Natl Acad Sci U S A. 2003;100(23):13722–7. 10.1073/pnas.2235214100. 14597698 10.1073/pnas.2235214100 PMC263880

[52] Tanaka M, Yamada E, Maekawa T, Ogata K, Takamiya N, Nakazono H, et al. Gender differences in subliminal affective face priming: a high-density ERP study. Brain Behav. 2021;11(4):e02060. 10.1002/brb3.2060. 33528111 10.1002/brb3.2060 PMC8035456

[53] Lee SA, Kim CY, Shim M, Lee SH. Gender differences in neural responses to perceptually invisible fearful face-an ERP study. Front Behav Neurosci. 2017;11:6. 10.3389/fnbeh.2017.00006. 28184189 10.3389/fnbeh.2017.00006 PMC5266704

[54] Prasad S, Mishra RK. The nature of unconscious attention to subliminal cues. Vision. 2019;3(3):38. 10.3390/vision3030038. 31735839 10.3390/vision3030038 PMC6802795

[55] Bell EC, Willson MC, Wilman AH, Dave S, Silverstone PH. Males and females differ in brain activation during cognitive tasks. Neuroimage. 2006;30(2):529–38. 10.1016/j.neuroimage.2005.09.049. 16260156 10.1016/j.neuroimage.2005.09.049

[56] Voyer D, Voyer SD, Saint-Aubin J. Sex differences in visual-spatial working memory: A meta-analysis. Psychon Bull Rev. 2017;24(2):307–34. 10.3758/s13423-016-1085-7. 27357955 10.3758/s13423-016-1085-7

[57] Lee BH, Richard JE, de Leon RG, Yagi S, Galea LAM. Sex differences in cognition across aging. Curr Top Behav Neurosci. 2023;62:235–84. 10.1007/7854_2022_309. 35467294 10.1007/7854_2022_309

[58] Rubia K, Hyde Z, Halari R, Giampietro V, Smith A. Effects of age and sex on developmental neural networks of visual-spatial attention allocation. Neuroimage. 2010;51(2):817–27. 10.1016/j.neuroimage.2010.02.058. 20188841 10.1016/j.neuroimage.2010.02.058

[59] Qian Y, Berenbaum SA, Gilmore RO. Vision contributes to sex differences in spatial cognition and activity interests. Sci Rep. 2022;12(1):17623. 10.1038/s41598-022-22269-y. 36271276 10.1038/s41598-022-22269-y PMC9586946

[60] Stoet G. Sex differences in the Simon task help to interpret sex differences in selective attention. Psychol Res. 2017;81(3):571–81. 10.1007/s00426-016-0763-4. 26957425 10.1007/s00426-016-0763-4 PMC5397428

[61] Nugent AC, Bain EE, Thayer JF, Sollers JJ, Drevets WC. Sex differences in the neural correlates of autonomic arousal: A pilot PET study. Int J Psychophysiol. 2011;80(3):182–91. 10.1016/j.ijpsycho.2011.03.001. 21414364 10.1016/j.ijpsycho.2011.03.001 PMC3091965

[62] Warthen KG, Boyse-Peacor A, Jones KG, Sanford B, Love TM, Mickey BJ. Sex differences in the human reward system: Convergent behavioral, autonomic and neural evidence. Soc Cogn Affect Neurosci. 2020;15(7):789–801. 10.1093/scan/nsaa104. 32734300 10.1093/scan/nsaa104 PMC7511890

[63] Wang J, Korczykowski M, Rao H, Fan Y, Pluta J, Gur RC, et al. Gender difference in neural response to psychological stress. Soc Cogn Affect Neurosci. 2007;2(3):227–39. 10.1093/scan/nsm018. 17873968 10.1093/scan/nsm018 PMC1974871

[64] Owens AP, Mathias CJ, Iodice V. Autonomic dysfunction in autism spectrum disorder. Front Integr Neurosci. 2021;15:787037. 10.3389/fnint.2021.787037. 35035353 10.3389/fnint.2021.787037 PMC8756818

[65] Williamson JB, Porges EC, Lamb DG, Porges SW. Maladaptive autonomic regulation in PTSD accelerates physiological aging. Front Psychol. 2014;5:1571. 10.3389/fpsyg.2014.01571. 25653631 10.3389/fpsyg.2014.01571 PMC4300857

[66] Guccione C, di Scalea GL, Ambrosecchia M, Terrone G, Di Cesare G, Ducci G, et al. Early signs of schizophrenia and autonomic nervous system dysregulation: A literature review. Clin Neuropsychiatry. 2019;16:2:86-97; https://www.ncbi.nlm.nih.gov/pubmed/34908942. PMC8662712 34908942

