# Supplementary figures and images for: Sex dependency of subconscious visual perception
Source: Biol Sex Differ. 2025 Oct 6;16:72. doi: 10.1186/s13293-025-00754-z (PMC12502163; doi:10.1186/s13293-025-00754-z)

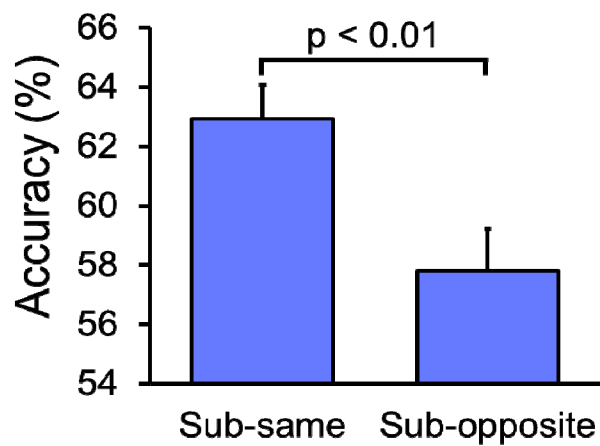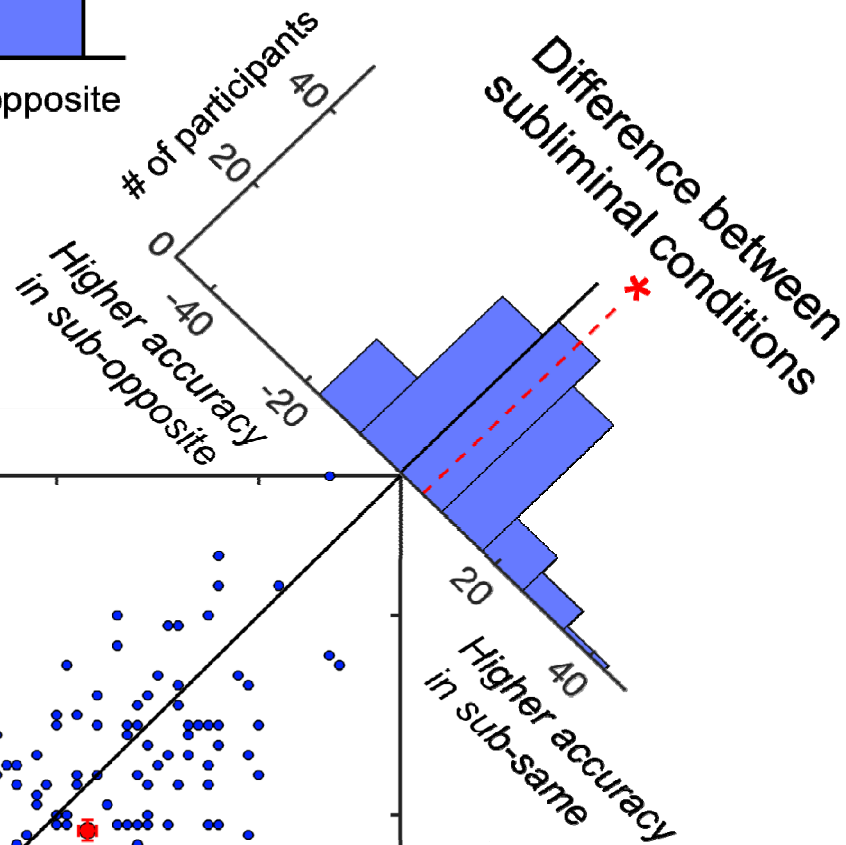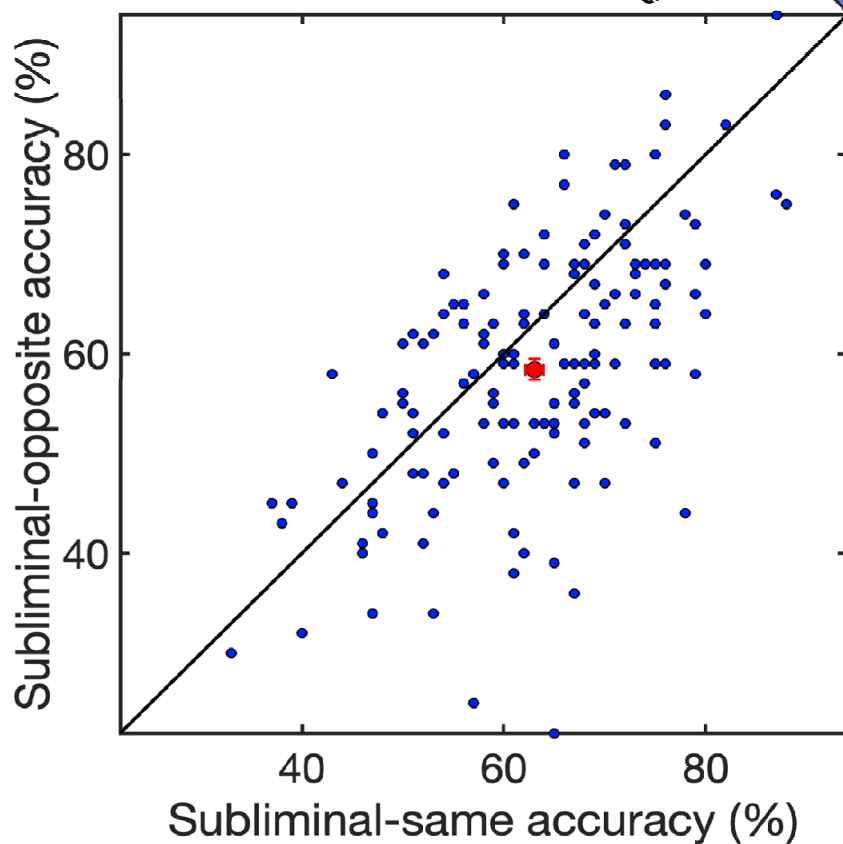

Supplement: Supplementary file 1 — Additional file 1. Supplementary Figure S1. Subliminal information influenced participants’ accuracy. The bar-graph shows the percentage of correct responses in Subliminal-same and Subliminal-opposite conditions. The p value indicates the main effect of the Information factor in the related ANOVA (when it was applied to the subliminal conditions). The scatterplot (below) displays individual participant accuracy data across both conditions, with the diagonal line indicating equal performance in both. Color-coded mean and standard error are included. The histogram (top right), scaled to the scatterplot, shows the distribution of accuracy differences between conditions, demonstrating a higher accuracy in the Subliminal-same condition. [file 13293_2025_754_MOESM1_ESM.pdf]

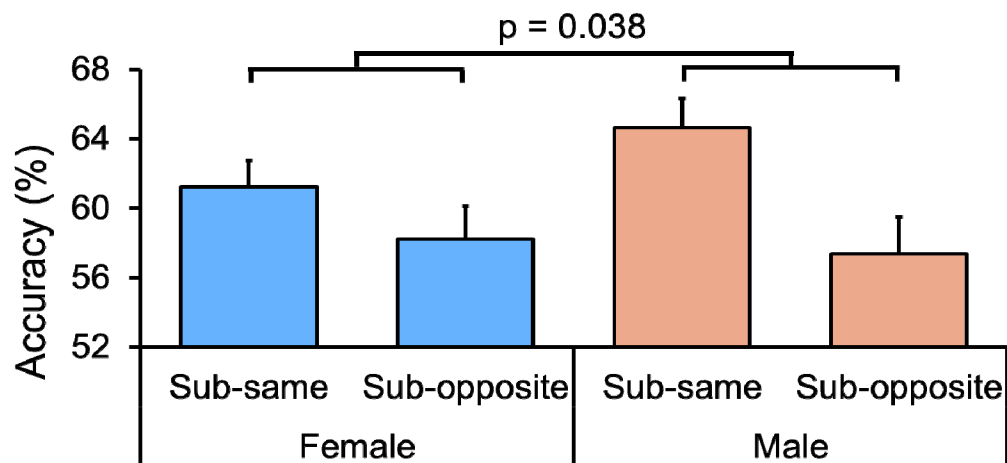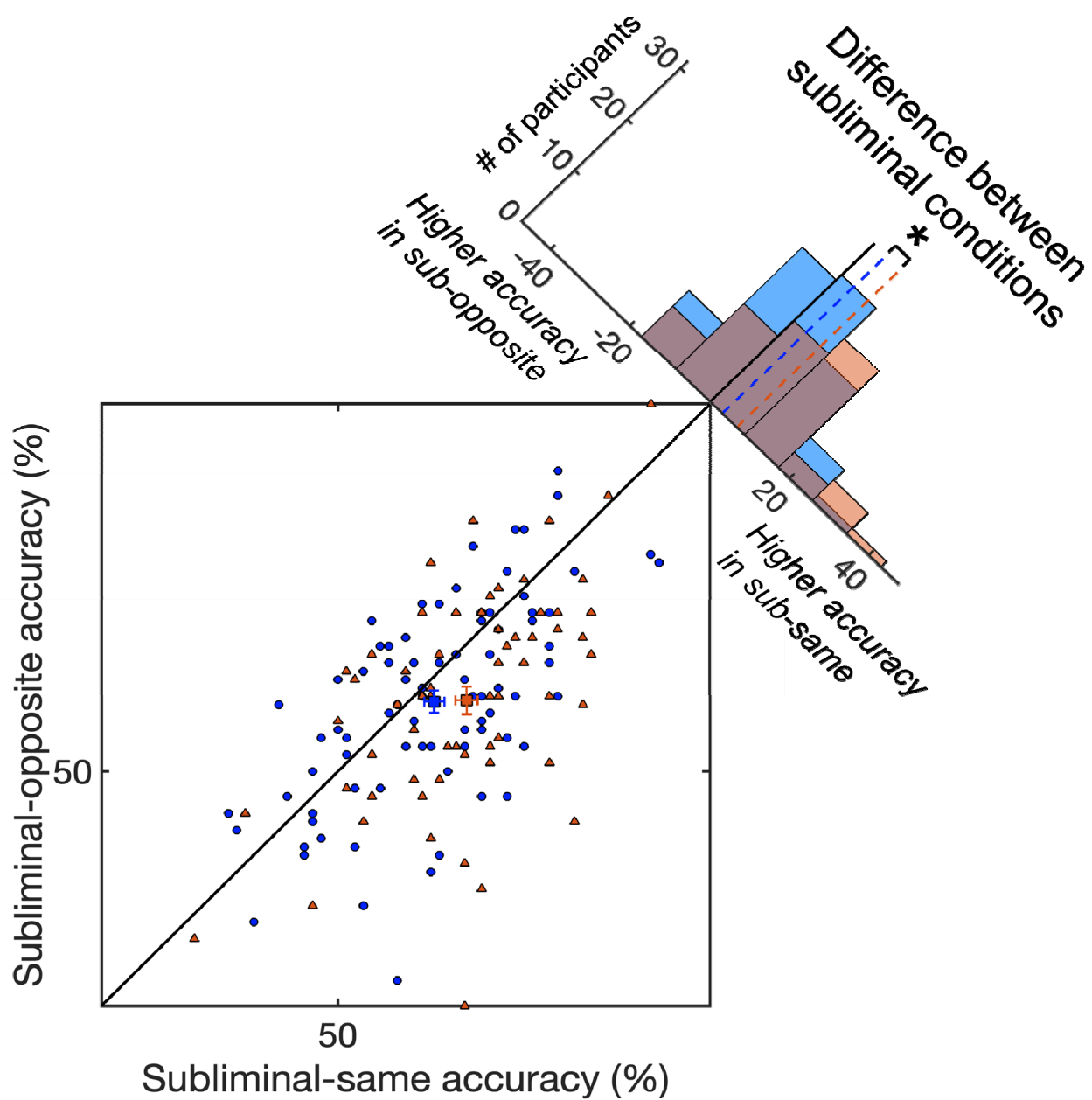

Supplement: Supplementary file 2 — Additional file 2. Supplementary Figure S2. Subliminal (subconscious) perception was sex dependent. (A) The bar-graph shows the percentage of correct responses in Subliminal-same and Subliminal-opposite conditions, separately for female and male participants. The p value indicates the significance of interaction between Information × Sex factors in the related ANOVA (when it was applied to the subliminal conditions). The scatterplot (bottom) shows individual participant accuracy across both conditions, for both females (blue circles) and males (orange triangles). Accuracy in the Subliminal-same condition was higher, particularly in males. [file 13293_2025_754_MOESM2_ESM.pdf]

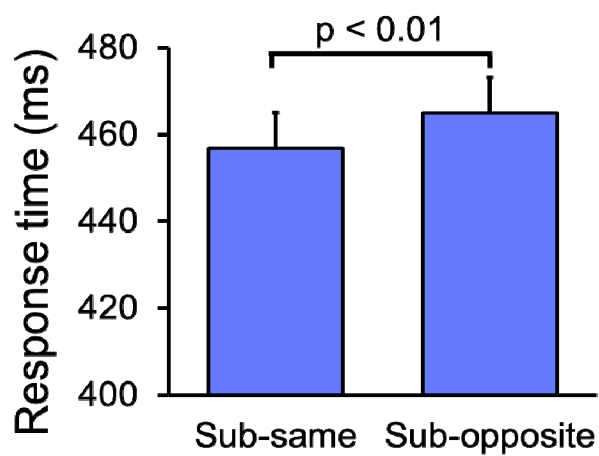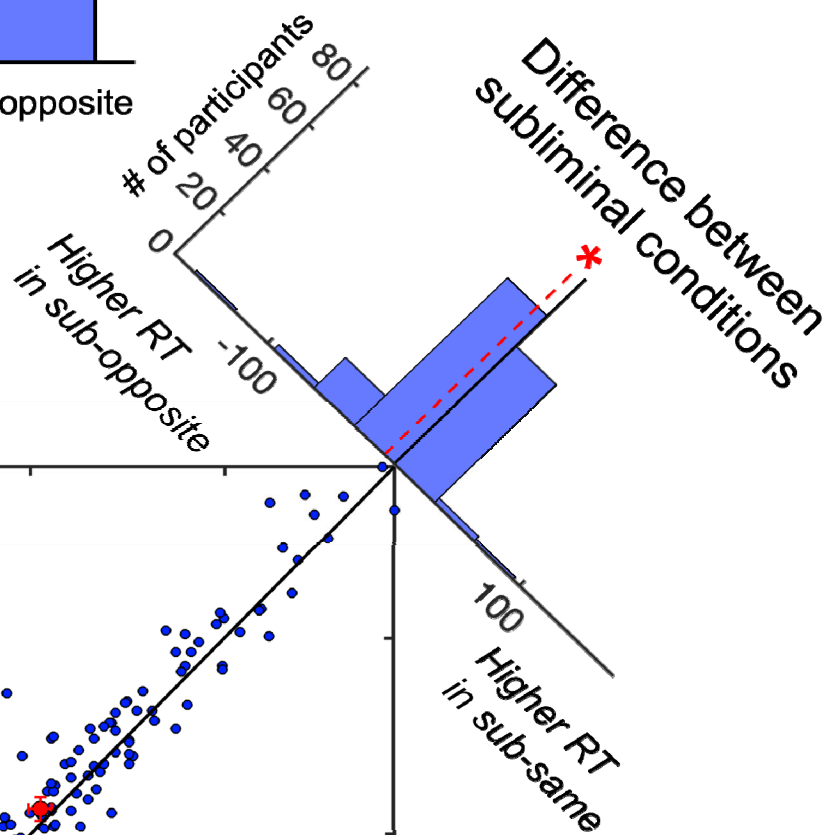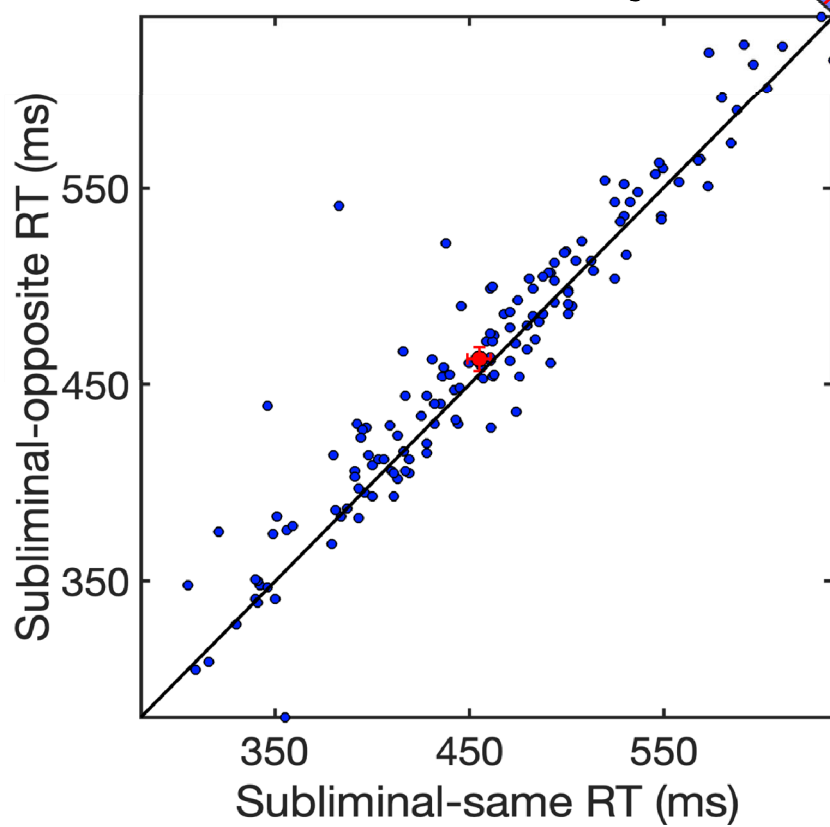

Supplement: Supplementary file 3 — Additional file 3. Supplementary Figure S3. Subliminal information influenced participants’ response time. The bar-graph shows the response time (RT) in Subliminal-same (Sub-same) and Subliminal-opposite (Sub-opposite) conditions. The scatterplot (below) displays individual participant RT data across both conditions, with the diagonal line indicating equal RT in both. Color-coded mean and standard error are included. The histogram (top right), scaled to the scatterplot, shows the distribution of RT differences between conditions, demonstrating a longer RT in the Subliminal-opposite condition. [file 13293_2025_754_MOESM3_ESM.pdf]

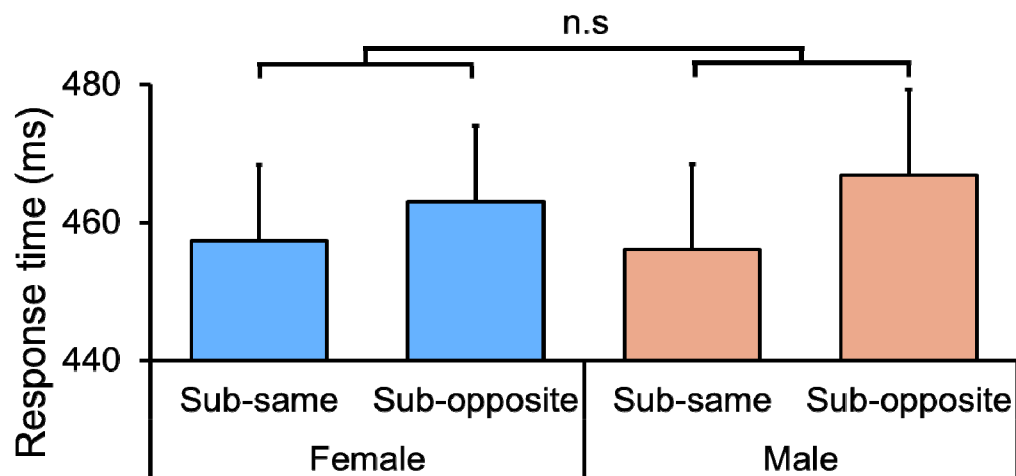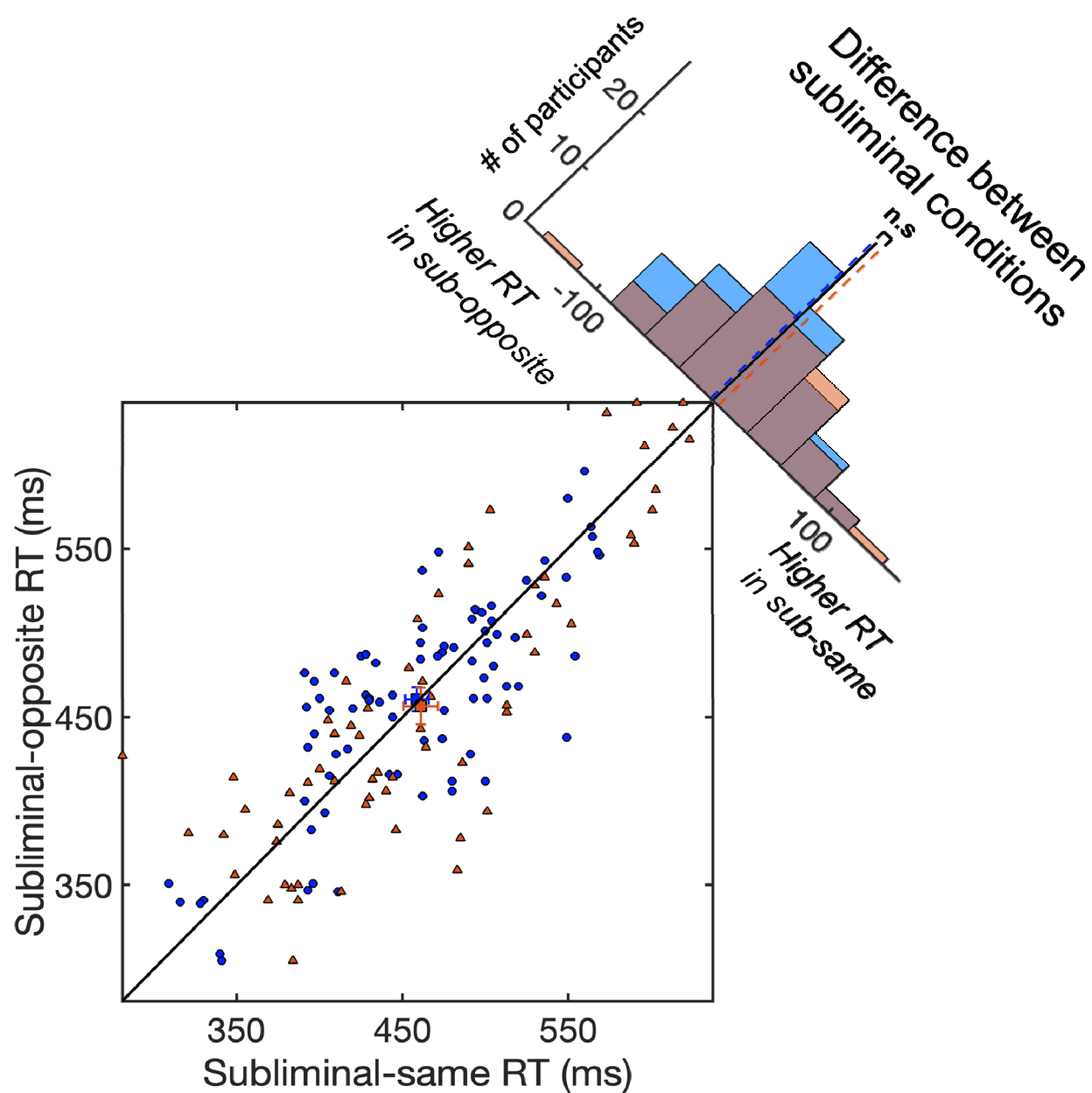

Supplement: Supplementary file 4 — Additional file 4. Supplementary Figure S4. No sex dependency was found in the effects of Subliminal information on participants’ response time. (A) The bar-graph shows the Response time (RT) in Subliminal-same and Subliminal-opposite conditions, separately for female and male participants. The p value indicates the significance of interaction between Information × Sex factors in the related ANOVA (when it was applied to the subliminal conditions).The scatterplot (bottom) shows individual participant RT across both conditions, for both females (blue circles) and males (orange triangles). [file 13293_2025_754_MOESM4_ESM.pdf]

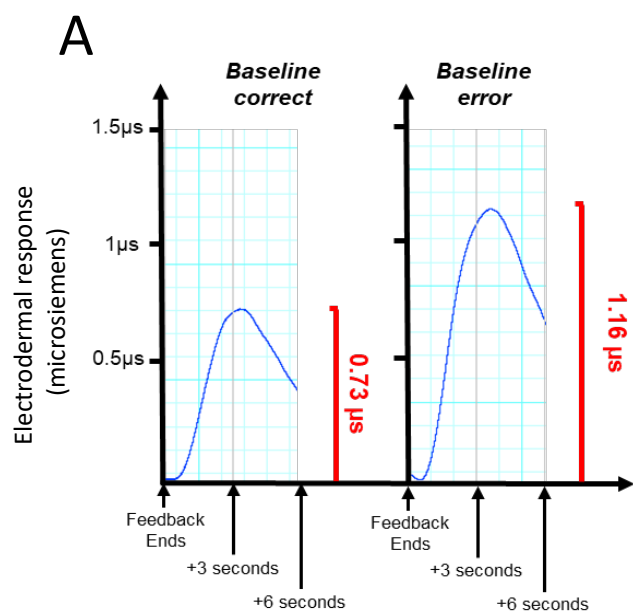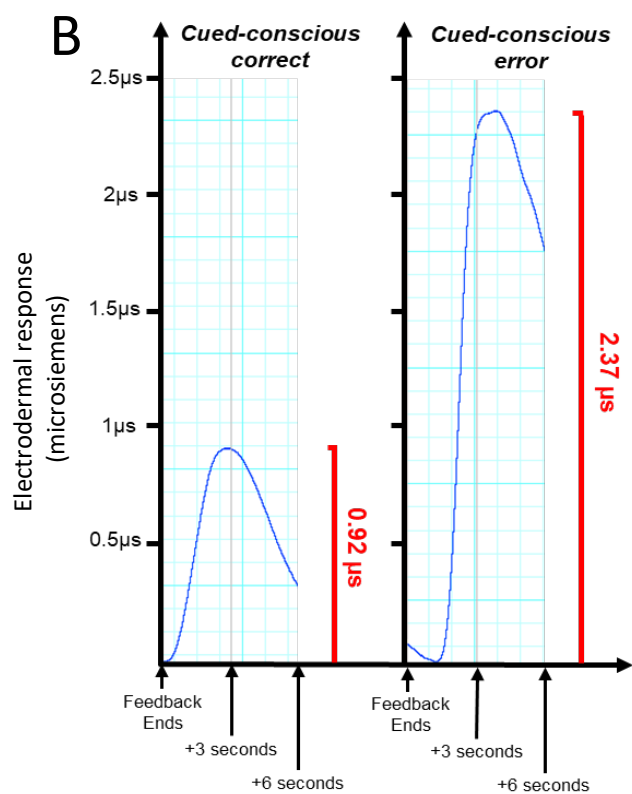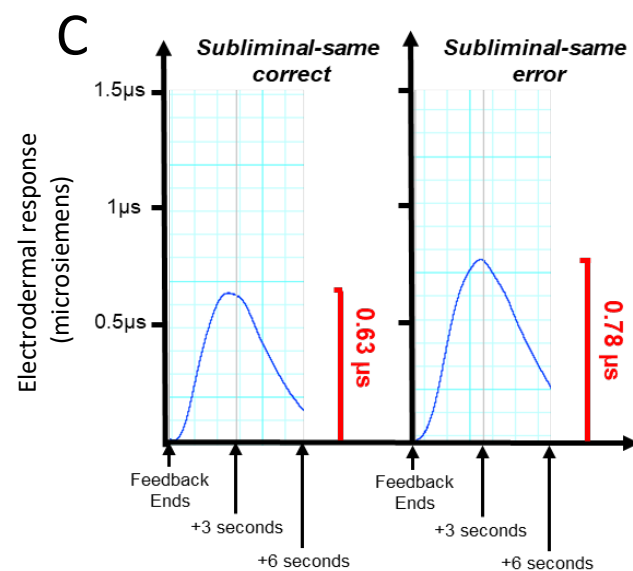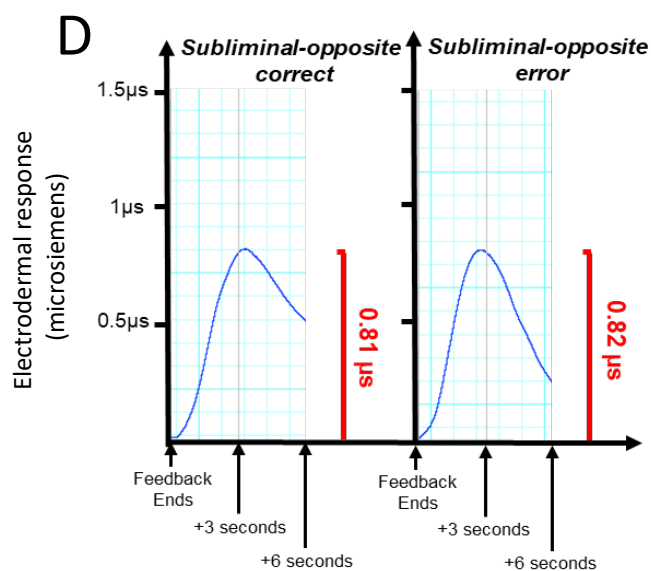

Supplement: Supplementary file 5 — Additional file 5. Supplementary Figure S5. Representative event-related EDA waveforms in supraliminal and subliminal conditions. (A-D) Representative event-related EDA waveforms in one participant present the phasic EDA responses (microsiemens) following feedback to correct (right panel) or error (left panel) in Baseline (A), Cued-conscious (B), Subliminal-same (C) and Subliminal-opposite (D) conditions. In all panels, the red vertical line indicates the difference between the minimum and maximum points (response amplitude) of the corresponding EDA waveforms. [file 13293_2025_754_MOESM5_ESM.pdf]
