# Supplementary material for: Sex dependency of subconscious visual perception
Source: Biol Sex Differ. 2025 Oct 6;16:72. doi: 10.1186/s13293-025-00754-z (PMC12502163; doi:10.1186/s13293-025-00754-z)
Supplement: Supplementary file 6 — Additional file 6. Supplementary Table 1. Behavioral and electrodermal activity values in females and males. Table 1 includes the mean ± SE for percentage of correct responses (% correct), Response time (RT) and normalised electrodermal activity for females and males. [file 13293_2025_754_MOESM6_ESM.pdf]

Supplementary Table 1							
	Female				*Male*		
	% correct	RT	Normalised EDA		% correct	RT	Normalised EDA
Baseline	60.33 ± 1.83	450.16 ± 11.53	0.94 ± 0.27		62.10 ± 2.06	455.70 ± 12.95	1.003 ± 0.30
Supraliminal	86.44 ± 1.75	306.78 ± 9.97	1.13 ± 0.061		84.94 ± 1.96	305.63 ± 11.20	1.07 ± 0.07
Subliminal-same	61.24 ± 1.53	457.36 ± 11.05	0.98 ± 0.04		64.63 ± 1.71	456.09 ± 12.41	0.97 ± 0.05
Subliminal-opposite	58.23 ± 1.90	462.97 ± 11.09	0.95 ± 0.03		57.38 ± 2.13	466.84 ± 12.45	0.96 ± 0.03
